# Photoreceptor engineering

**DOI:** 10.3389/fmolb.2015.00030

**Published:** 2015-06-17

**Authors:** Thea Ziegler, Andreas Möglich

**Affiliations:** ^1^Biophysikalische Chemie, Institut für Biologie, Humboldt-Universität zu BerlinBerlin, Germany; ^2^Lehrstuhl für Biochemie, Universität BayreuthBayreuth, Germany

**Keywords:** allostery, light, optogenetics, protein engineering, sensory photoreceptor, signal transduction

## Abstract

Sensory photoreceptors not only control diverse adaptive responses in Nature, but as light-regulated actuators they also provide the foundation for optogenetics, the non-invasive and spatiotemporally precise manipulation of cellular events by light. Novel photoreceptors have been engineered that establish control by light over manifold biological processes previously inaccessible to optogenetic intervention. Recently, photoreceptor engineering has witnessed a rapid development, and light-regulated actuators for the perturbation of a plethora of cellular events are now available. Here, we review fundamental principles of photoreceptors and light-regulated allostery. Photoreceptors dichotomize into associating receptors that alter their oligomeric state as part of light-regulated allostery and non-associating receptors that do not. A survey of engineered photoreceptors pinpoints light-regulated association reactions and order-disorder transitions as particularly powerful and versatile design principles. Photochromic photoreceptors that are bidirectionally toggled by two light colors augur enhanced spatiotemporal resolution and use as photoactivatable fluorophores. By identifying desirable traits in engineered photoreceptors, we provide pointers for the design of future, light-regulated actuators.

## Introduction

Ever since the original description of algal channelrhodopsins as light-gated ion channels (Nagel et al., 2002, 2003), various sensory photoreceptors have been widely deployed across biology as light-regulated actuators for precise perturbation and probing of cellular events. The underlying approach, dubbed optogenetics (Deisseroth et al., 2006), derives its power and versatility from several key properties of sensory photoreceptors: first, they can be genetically encoded and functionally expressed *in situ*, thus affording exquisite molecular targeting and superior spatial resolution compared to conventional perturbation, e.g., by electrical or chemical means; second, they can be triggered by light that penetrates living matter to certain depth, thus affording non-invasive control and superior temporal resolution; and third, they operate reversibly, thus affording transient and repeat perturbation. While initial optogenetic applications were restricted to the neurosciences and exclusively relied on light-gated ion channels and light-driven ion pumps, the concept clearly transcends ion transport and applies to a plethora of biologically relevant processes. Early on, optogenetics solely utilized naturally occurring sensory photoreceptors, but customized photoreceptors have since been engineered that achieve light perturbation of numerous cellular events of interest as diverse as transcription, enzyme catalysis, protein degradation, and cell motility.

Here, we survey the engineering and optogenetic application of sensory photoreceptors. The sheer number of engineered photoreceptors testifies to the vigor of the field, but it effectively precludes a detailed discussion of each and every example. Rather than providing a mere enumeration, which—given the pace of current developments—would be outdated fairly soon, we discuss general aspects and strategies by means of particularly illustrative examples. By identifying recurring and overarching features, we aspire to provide a guide for the selection of photoreceptors for optogenetic applications as well as for their engineering. By necessity, this treatise emphasizes certain areas less than others, and we refer the reader to review articles for coverage of natural photoreceptors (Möglich et al., 2010b), protein engineering (Moffat et al., 2013; Pudasaini et al., 2015; Schmidt and Cho, 2015; Shcherbakova et al., 2015), and applications in cell biology (Beyer et al., 2015b; Fan and Lin, 2015; Zhang and Cui, 2015) and neuroscience (Pashaie et al., 2014).

## Photoreceptor fundamentals

**Figure 1:**
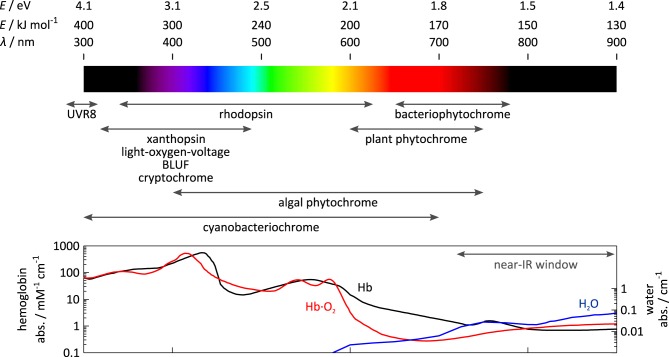
**Spectral sensitivity of photoreceptors**. The spectral sensitivity of sensory photoreceptors ranges from the UV to the near-infrared region of the electromagnetic spectrum. Visible light is strongly absorbed or scattered by opaque biological tissue owing to the presence of lipids, heme-containing proteins, e.g., hemoglobin (Hb), and other pigments. Above 700 nm within the so-called near-infrared window, scattering and absorption by pigments are lower, and light penetrates biological tissue more deeply.

To convert light signals, i.e., electromagnetic waves, into cellular signals, i.e., “jiggling and wiggling of atoms and molecules” (Feynman, 1963), sensory photoreceptors harbor two principal functions in dedicated modules: first, a photosensor module absorbs light; second, an effector module exerts biological activity (e.g., ion transport, catalysis, protein interaction). The engineering of photoreceptors with emergent properties encompasses the modification of photosensors and/or effectors as well as the modular recombination/rewiring of photosensors and effectors. Depending upon whether these modules are realized as distinct proteins, distinct protein domains or fragments of a single protein domain, physical separation and subsequent recombination of photosensors and effectors may be more or less challenging (cf. Section Allostery of Photoreceptors). Each photosensor bears an organic chromophore that contains a conjugated π electron system, that is either covalently or non-covalently bound, and that derives from either amino-acid side chains or small-molecule metabolites. Based on identity and photochemistry of their chromophores, photoreceptors distribute into several classes with different spectral sensitivities as indicated in Figure 1. For example, plant UV-B receptors, exemplified by *Arabidopsis thaliana* UVR8, use tryptophan side chains to sense UV-B light; the flavin-nucleotide-binding cryptochromes, LOV (light-oxygen-voltage) and BLUF (blue-light sensors using flavin-adenine dinucleotide) proteins are sensitive to blue light; within the rhodopsins, individual representatives use retinal to respond to different light bands in the UV to red spectral region; phytochromes (Phys) from terrestrial plants employ linear tetrapyrroles (bilins) to achieve sensitivity toward red and near-infrared light, but a recently characterized lineage of algal phytochromes essentially covers the entire visible and near-infrared spectrum (Rockwell et al., 2014); similarly, cyanobacteriochromes (CBCRs) use bilins as well and display increased spectral diversity covering the electromagnetic spectrum from UV to near-infrared wavelengths.

The choice of photoreceptor for optogenetic application is dictated by at least two principal considerations (also cf. Section Guidelines for Photoreceptor Engineering), namely chromophore availability and tissue penetration of light. First, to achieve true genetic encoding, not only must a photoreceptor fold autonomously and incorporate its chromophore, but also the chromophore must be available in the target tissue to sufficient extent. While the former is generally true, the latter is only the case for certain chromophores. Due to their role as core cofactors in diverse metabolic proteins and enzymes, flavin nucleotides universally occur in different cells and organisms. Moreover, the widespread, successful deployment of rhodopsin-based ion channels and pumps in diverse contexts testifies that this also pertains to retinal in many tissues (Deisseroth et al., 2006; Karunarathne et al., 2013). Several recent studies indicate that the oxidized linear tetrapyrrole biliverdin (BV) also recurs in commonly used model systems (Piatkevich et al., 2013a; Gasser et al., 2014; Ryu et al., 2014), presumably as a degradation product of heme. By contrast, reduced tetrapyrroles, such as phycocyanobilin (PCB), that plant Phys and CBCRs depend on, are not generally available in most systems of optogenetic interest. Second, for efficient optical perturbation *in situ*, light of the required wavelength must penetrate tissue to sufficient depth. While light penetration often is of little concern for studies in microorganisms or cell culture where the relevant dimensions are small, it may become limiting in applications in deep tissue or whole animals. Due to light scattering in opaque tissues, in particular by lipids (Chung et al., 2013), and absorption by heme-containing proteins and other pigments, tissue penetration is severely limited across essentially the entire visible spectrum; however, within the spectral region above 700 nm, often denoted the “near-infrared spectral window,” high penetration depths are achieved. The spectral sensitivity of certain photoreceptor classes, e.g., LOV, BLUF, cryptochrome, and UVR8, is largely invariant between representatives, essentially owing to the rigid scaffold of their chromophores. By contrast, individual representatives of the rhodopsins (Ernst et al., 2014), phytochromes (Rockwell et al., 2014), and cyanobacteriochromes (Ikeuchi and Ishizuka, 2008; Rockwell and Lagarias, 2010) considerably differ in their spectral sensitivities which can be accounted for by the structural malleability and varying protonation of their chromophores, as well as by electrostatic interactions with residues lining the chromophore-binding pocket. As a corollary, these photoreceptors are amenable to so-called color tuning, the variation of spectral sensitivity via mutagenesis. However, color tuning is often fraught with problems of limited predictability and inadvertent effects on signal transduction (cf., e.g., Ernst et al., 2014).

**Figure 2:**
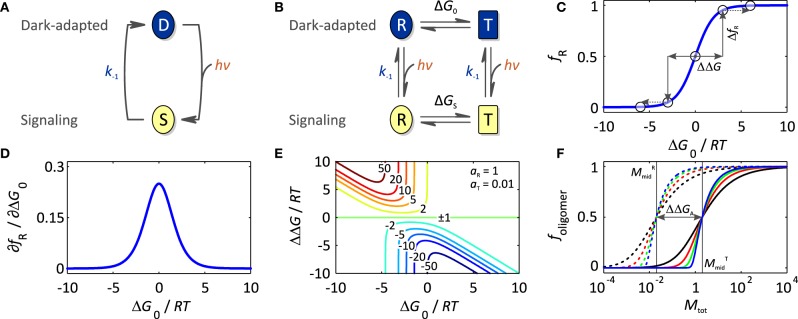
**Thermodynamics of photoreceptors**. **(A)** Absorption of light drives the transition from the dark-adapted state D to the signaling state S, which depending upon photoreceptor, may involve reaction intermediates. As the life time of these intermediates is usually much smaller than the time scale of optogenetic applications, operationally the reaction is considered unimolecular and single-step even though mechanistically it may not be. Once in the signaling state, the photoreceptor thermally reverts to the dark-adapted state which again is assumed to be unimolecular, single-step with rate constant *k*_−1_. **(B)** In the framework of an allosteric model, signal receptors exist in dynamic equilibrium between states R (relaxed) of higher biological activity *a*_R_ and T (tense) of lower biological activity *a*_T_. In the dark, the equilibrium between R and T is governed by the free energy difference, Δ*G*_0_, between these states. Introduction of a signal, light in case of sensory photoreceptors, (de)stabilizes the R and T states, and thus shifts the equilibrium to Δ*G*_S_. **(C)** The fractional population of the R state, *f*_R_, is a function of Δ*G*_0_. Significant populations of R and T coexist close to equilibrium, i.e., Δ*G*_0_ = 0. Free energy perturbations ΔΔ*G*, e.g., introduced by signal or mutation, shift the equilibrium to a new value (arrows), where positive values of ΔΔ*G* increase *f*_R_, and negative values of ΔΔ*G* decrease *f*_R_. **(D)** *f*_R_ is maximally sensitive to free energy perturbations near Δ*G*_0_ = 0 but essentially invariant if |Δ*G*_0_| >> 0. **(E)** The dynamic range *d* for the signal response, i.e., the ratio of the biological activities in the absence and presence of signal, depends on the initial equilibrium between R and T (Δ*G*_0_), the magnitude of the perturbation (ΔΔ*G*), and the intrinsic activities of R and T, *a*_R_ and *a*_T_. Contour lines denote the magnitude of *d* for the parameter set *a*_R_ = 1 and *a*_T_ = 0.01, where positive values of *d* refer to light-activated and negative values to light-repressed photoreceptors. **(F)** Isotherms for homo-association of photoreceptors where *f*_oligomer_ denotes the protein fraction in oligomeric form and *M*_tot_ the total monomer concentration. Black, red, green and blue curves refer to dimeric, trimeric, pentameric and decameric complexes, respectively, bold lines correspond to the low-affinity T state and dashed lines to the high-affinity R state. For photoassociating systems, ΔΔ*G*_A_ is positive, and light promotes a transition from the low-affinity (solid) to the high-affinity (dashed) isotherms. *Vice versa*, for photodissociating systems, ΔΔ*G*_A_ is negative, and light promotes a transition from high-affinity to low-affinity isotherms.

In the absence of light, photoreceptors adopt their thermodynamically most stable, dark-adapted state, denoted D (cf. Section Thermodynamics of Photoreceptors). Upon light absorption, they undergo a so-called photocycle, a series of photochemical reactions within the chromophore and accompanying structural and dynamic transitions within the surrounding protein scaffold (Figure 2A). In addition to D, the photocycle minimally comprises the signaling state S which persists for milliseconds to many hours depending upon photoreceptor and which differs from D in structure, dynamics and function. For an in-depth treatise of photochemistry we refer to pertinent review articles (Hegemann, 2008; Möglich et al., 2010b). While the molecular aspects widely differ across classes, in all photoreceptors the initial photochemical reaction toward formation of the signaling state S is very fast, e.g., bond isomerization or inter-system crossing, so as to achieve high quantum yields for photoreception, at the same time minimizing competing reactions, i.e., chiefly fluorescence and internal conversion. The initial reaction may be succeeded by additional, slower steps, e.g., formation of a covalent bond in LOV proteins (Conrad et al., 2014), but in all cases the signaling state S is formed after photon absorption within at most microseconds which is faster than the timescale of most physiological responses. For the purpose of this overview article, we thus resort to a grossly simplified, operational model of the photocycle in which the light-driven reaction to the signaling state is considered unimolecular and single-step with rate constant *k*_1_(*I*) depending upon light intensity *I*. A thermally driven, spontaneous reaction, denoted dark reversion, closes the photocycle and reverts S back to D; for simplicity, we consider this reversion as unimolecular and single-step with rate constant *k*_−1_.

The absolute light sensitivity of a photoreceptor is determined by its absorption cross section and its overall quantum yield for formation of the signaling state, lumped together in the rate constant *k*_1_(*I*). However, optogenetic experiments are often conducted under constant illumination at photostationary state where there is no net change in the populations of D and S, i.e., where the velocities of the reverse reaction *v*_−1_ and the light-driven forward reaction *v*_1_ equal. The balance between D and S at photostationary state is governed by the effective light sensitivity of the photoreceptor, i.e., the ratio between the forward and reverse rate constants *k*_1_(*I*) and *k*_−1_, the former of which is a function of applied light intensity *I*. In optogenetic applications, the response kinetics of light-sensitive systems are often of key relevance, i.e., how soon after onset of illumination the desired biological effect manifests (*on*-kinetics), and how long after stop of illumination the effect persists (*off*-kinetics). With the exception of fast events in the neurosciences, the intrinsic photochemical response of photoreceptors after initial photon absorption is near-instantaneous on most biologically relevant time scales and thus not limiting; rather, *on*-kinetics are often limited by the light dose that can be delivered per unit of time to the system under study which is subject to instrumental (e.g., maximum output of light sources, tissue penetration of light) and biological constraints (e.g., radiation damage, phototoxicity). Moreover, biological steps subsequent to photochemical events may be rate-limiting; in particular, certain cellular responses, notably gene expression, are inherently slow. *Off*-kinetics are governed by the rate constant for dark recovery *k*_−1_ at physiological conditions as well as by slow biological processes. Note that dark-recovery reactions are often associated with large activation energies and can hence be strongly temperature-dependent. Photochromic photoreceptors, which we discuss in detail in Section Photochromic Photoreceptors, offer potentially much faster *off*-kinetics via active, light-driven reversion of the signaling state to the dark-adapted state. For some photoreceptor classes, specifically LOV proteins, certain rhodopsins and phytochromes, mutations have been identified that greatly accelerate or decelerate the dark-recovery reaction and that could hence be used to modulate *off*-kinetics (Yang et al., 2007; Berndt et al., 2009; Zoltowski et al., 2009). Less is known on mutations that would modify the forward kinetics (*k*_1_); however, to achieve sensitive signal reception, photoreceptors generally possess high quantum yields for formation of the signaling state and hence there is little, if any, scope for further enhancement. Further, any variation of forward or reverse kinetics will invariably affect the effective light sensitivity at photostationary state (cf. above). Finally, mutations introduced with the intent of modulating photocycle kinetics may have inadvertent, often deleterious effects on signal transduction, even to the extent of completely abolishing any downstream response (Diensthuber et al., 2014).

## Thermodynamics of photoreceptors

**Figure 3:**
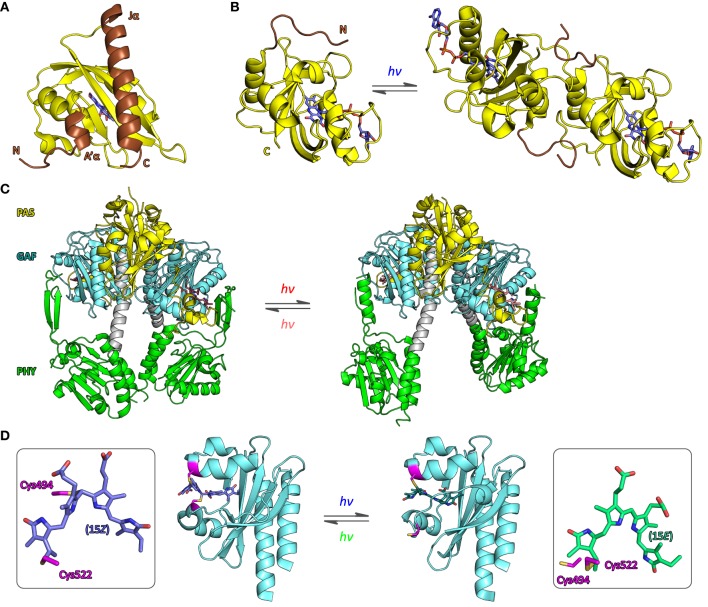
**Structure of photoreceptors**. **(A)** The LOV2 domain of *Avena sativa* phototropin 1 (*As*LOV2) adopts the canonical PAS fold (Möglich et al., 2009b) with N-terminal and C-terminal helices, denoted A′α and Jα (brown), packed onto the outer face of a five-stranded antiparallel β sheet (PDB entry 2V0U, Halavaty and Moffat, 2007). Light absorption promotes unfolding and concomitant dissociation of Jα from the LOV core domain (Harper et al., 2003). **(B)** The structures of the *Neurospora crassa* LOV protein Vivid in its dark-adapted state (left, 2PD8, Zoltowski et al., 2007) and in its signaling state (right, 3RH8, Vaidya et al., 2011) provide an atomic perspective on refolding of an N-terminal extension to the LOV core domain (brown) and concomitant dimerization. **(C)** High-resolution structures of the photosensor module of *Deinococcus radiodurans* bacteriophytochrome, comprising PAS, GAF, and PHY domains, in its dark-adapted Pr state (left, 4O0P) and red-light-adapted Pfr state (right, 4O01, Takala et al., 2014) implicate a pivot motion and splaying apart of the PHY domains as the molecular mechanism for light-induced signal transduction. **(D)** The structure of a cyanobacteriochrome photosensor from the PixJ protein of *Thermosynecchococcus elongatus* BP-1 has been determined in the dark-adapted, blue-light-absorbing 15*Z* state (left, 4FOF, Burgie et al., 2013) and the light-adapted, green-light-absorbing 15*E* state (right, 3VV4, Narikawa et al., 2013), where *Z* and *E* refer to isomers of the C15=C16 double bond of the PCB chromophore that is covalently bound to cysteine 522 (see boxes). In the 15*Z* state cysteine 494 (magenta) forms a second thioether bridge to the C10 atom of the chromophore.

Before regarding specific case studies, it is illuminating to consider the general thermodynamics that govern signaling processes and set energetic limits on both natural and engineered photoreceptors. Adapting concepts from classic models of allostery (Monod et al., 1965; Wyman and Gill, 1990; Motlagh et al., 2014), we previously introduced a simple framework for stimulus perception in signal receptors (Möglich et al., 2009b; Möglich and Moffat, 2010) (Figure 2A). Within this model, signal receptors are assumed to be in dynamic equilibrium between states of lower (T, tense) and higher (R, relaxed) biological activity. In the dark-adapted state D, the equilibrium *L*_0_ = [*T*]_0_/[*R*]_0_ is determined by the free energy difference Δ*G*_0_ = −*RT* ln *L*_0_ between these states. Introduction of signal (de)stabilizes T and R to different extents, thus alters the free energy difference by ΔΔ*G* to Δ*G*_S_, and shifts the equilibrium to the new value *L_S_* = [*T*]_*S*_/[*R*]_*S*_ in the signaling state S. Consequently, signal does not alter the pre-existing, intrinsic states themselves but shifts the dynamic equilibrium between them. Evidence in support of co-existing states of different biological activity has been obtained for both signal receptors in general, e.g., (Volkman et al., 2001), and photoreceptors in specific (e.g., Yao et al., 2008). In particular, the widely deployed LOV2 domain from *Avena sativa* phototropin 1 (*As*LOV2, Figure 3A) possesses a C-terminal extension, denoted Jα, that exists in conformational equilibrium between an α-helical state docked against the LOV core (T state) and an unfolded, undocked state (R state) (Harper et al., 2003; Halavaty and Moffat, 2007). In the dark, the equilibrium between the coexisting states is shifted toward the folded, helical state, but under blue light the unfolded, undocked state predominates (Yao et al., 2008). An N-terminal helical extension, denoted A′α, also contributes to the light-promoted unfolding reaction (Zayner et al., 2012). In general, if the absolute free energy difference between states T and R is large, the equilibrium is almost entirely shifted to one side, and the minority state may not be detectable at all (Figure 2B). When applied to photoreceptors, the allosteric model can elegantly account for both light-activated and light-repressed phenomena. For light-activated proteins, ΔΔ*G* is positive, and the fraction of the more active R state, *f*_R_, increases in the signaling state relative to that in the dark-adapted receptor; for light-repressed proteins, ΔΔ*G* is negative, and *f*_R_ is diminished in the signaling state (Figure 2C). The fraction of R state is maximally sensitive to signal-induced free energy perturbations near equilibrium, i.e., Δ*G*_0_ = 0 (Figures 2C,D); by contrast, if |Δ*G*_0_| >> 0, free energy perturbations will have less effect on *f*_R_. However, as also noted by Schmidt and Cho (2015), applications of sensory photoreceptors more often do not demand maximum sensitivity of *f*_R_ but rather maximum dynamic range *d*, here defined as the ratio of biological activities in the dark-adapted and in the signaling states, where positive values of *d* denote light activation and negative values denote light repression. The activities in the dark-adapted and signaling states in turn are given by the relative fractions *f*_T_ and *f*_R_ in the D and S states as well as by the elementary activities *a*_T_ and *a*_R_ of the T and R states, respectively. The ratio *a*_R_/*a*_T_ defines an upper limit of the maximum dynamic range that can be achieved. Furthermore, high dynamic ranges of light activation (*d* >> 1) can only be realized if the dark-adapted photoreceptor predominantly assumes the T state (Figure 2E), i.e., Δ*G*_0_ < 0. Likewise, high dynamic ranges for light repression (*d* << −1) can only be achieved if the photoreceptor in its signaling state predominantly populates T, i.e., Δ*G*_S_ < 0. Put another way, the magnitude of *d* is mainly dependent upon how well biological activity can be shut off in the low-activity state, i.e., in the dark-adapted state for light-activated receptors and in the signaling state for light-repressed receptors. Notably, these considerations directly bear on signal transduction and the engineering of photoreceptors: although it may be challenging to much alter ΔΔ*G*, i.e., the amount of free energy that can be derived from light perception, it is well documented that Δ*G*_0_ can deliberately be changed, e.g., via site-directed mutagenesis, so as to achieve improved dynamic range (Strickland et al., 2010).

Dating back to the original implementation by Quail and coworkers (Shimizu-Sato et al., 2002), photoreceptors that undergo association/dissociation reactions in response to light absorption have been widely deployed in the engineering of light-responsive systems and in optogenetics. Their widespread prevalence, their undisputed success in optogenetics, and the often fairly predictable engineering strategies warrant a specific spotlight on these associating photoreceptors (cf. Section Associating Photoreceptors and Optogenetic Applications). Signal-dependent oligomerization reactions can be rationalized by a lower dissociation constant *K*^R^_D_ in the R state, i.e., higher affinity, than in the T state, *K*^T^_D_ (Figure 2F). Of key importance, the transition between dark-adapted and signaling states hence involves changes in oligomeric state, meaning that the system response to light strongly depends upon the concentration of photoreceptor molecules. As determined by the magnitude of *K*_D_, at a total photoreceptor concentration *M*_tot_, a fraction of molecules *f*_oligomer_ will be in oligomeric complex, and the remaining fraction of 1-*f*_oligomer_ will be present as monomers. A number of fundamental insights can be gleaned from inspection of the corresponding association isotherms (Figure 2F). First, with increasing number *n* of monomers in the oligomeric complex, the association reaction becomes increasingly cooperative, and the isotherm between monomeric and oligomeric state becomes steeper. Second, the total photoreceptor concentration *M*_tot_ needs to be matched to the affinities in both the T and R states; notably, the midpoint of the isotherms, where the fraction of molecules in monomeric form equals that in oligomeric complex, occurs at $M_{\text{mid}}=2\cdot \sqrt[{n-1}]{K_{\text{D}}/n}$. As a consequence, significant light-dependent changes in oligomeric state can only be induced if *M*_tot_ ranges between *M*^R^_mid_ and *M*^T^_mid_. By contrast, if *M*_tot_ << *M*^R^_mid_, no oligomeric complex will be formed, even in the higher-affinity state R; if *M*_tot_ >> *M*^T^_mid_, photoreceptor molecules will be largely present in oligomeric complex in both the T and R states. Third, variations of *M*_tot_ near either *M*^T^_mid_ or *M*^R^_mid_ can have disproportionate effect on the degree of oligomerization and extent of regulation by light. For example, such variations may arise from differing protein levels across expression systems and/or mutant variants of photoreceptors. Fourth, the extent of photoreceptor activation can have immense influence on the cooperative association reaction. Drastic changes in oligomerization occur for concentrations of light-activated molecules in the signaling state around the threshold defined by *M*^R^_mid_; the degree of oligomerization and the system response can thus depend on applied light dose in highly non-linear manner. Fifth, to induce by signal a change in *M*_mid_ of a factor *x*, a total free energy perturbation of ΔΔ*G_A_* = (*n* − 1)·RT ln *x* is required, or (*n* − 1)/*n* · *RT* ln *x* per monomer. In the example in Figure 2F, *M*^T^_mid_ and *M*^R^_mid_ differ by a 100-fold, corresponding to about 11.4 kJ mol^−1^ per monomer for large *n*.

Due to space constraints, we only consider fully cooperative homo-association reactions, i.e., cases where the Hill coefficient amounts to *n*. Equations can also be obtained for less cooperative reactions and hetero-association reactions; fundamentally similar considerations hold for these scenarios as well.

## Allostery of photoreceptors

**Figure 4:**
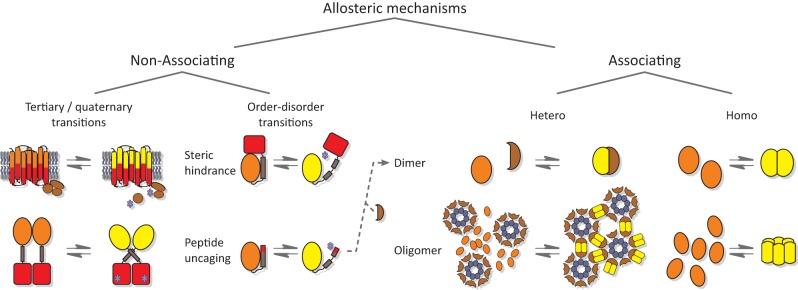
**Allostery of photoreceptors**. Allosteric signal transduction mechanisms realized in natural and engineered photoreceptors are grouped into non-associating and associating categories. Within the category of non-associating photoreceptors (Section Non-associating Photoreceptors and Optogenetic Applications), a disparate subclass of receptors relies on light-induced transitions of tertiary and quaternary (other than association) structure to regulate biological activity (Section Light-regulated Tertiary and Quaternary Structural Transitions). A second subclass capitalizes on light-induced order-disorder transitions, epitomized by Jα helix unfolding in *As*LOV2 (Harper et al., 2003), most often to restrict access to either an effector active site or to a peptide epitope in light-regulated manner (Section Light-regulated Order-disorder Transitions). Within the category of associating photoreceptors (Section Associating Photoreceptors and Optogenetic Applications), light-induced signal transduction goes along with a change in oligomeric state. Association can be either light-promoted or light-repressed; can be between alike (homo) or different (hetero) partners; and can be of dimeric or higher stoichiometry. Note that other categorization schemes are conceivable as well; in particular, several photoreceptors based on light-induced Jα unfolding subsequently undergo heteroassociation (Lungu et al., 2012; Strickland et al., 2012; Guntas et al., 2015) (indicated by dashed arrow). Orange and yellow symbols denote T and R states, respectively, of photosensor modules; red symbols denote effector modules; and blue stars indicate effectors in their high-activity state.

Numerous studies of natural photoreceptors have unveiled ingenious allosteric strategies by which light signals are translated into changes of biological activity. Many of these strategies have subsequently been co-opted for photoreceptor engineering. Invariably, initial light-induced changes within the chromophore-binding pocket of the photosensor have to be propagated and allosterically coupled to associated, often distal effector modules. When present as isolated modules, photosensors and effectors usually retain their elementary functions of absorbing light and exerting biological activity, respectively. By contrast, the desired property of light-regulated biological activity is only accomplished in composite photoreceptors where photosensor and effector are linked in a manner conducive to thermodynamic coupling between these modules. Consequently, the physical nature of the linker (topology, length, sequence, structure, dynamics) between photosensor and effector modules clearly is of central importance for light-regulated allostery. In fact, successful photoreceptor engineering has often amounted to optimizing the inter-module linker, whereas photosensor and effector have been minimally modified or left untouched altogether (cf. below). We categorize the plethora of engineering examples to date (Figure 4) based on whether photoreception involves light-regulated association (Section Associating Photoreceptors and Optogenetic Applications, **Table 2**) or not (Section Non-associating Photoreceptors and Optogenetic Applications, Table 1). It should be noted that the below strategies mostly represent specific manifestations of more general design concepts that have proven successful in the engineering of allosterically regulated, mostly light-inert proteins (Makhlynets et al., 2015; Stein and Alexandrov, 2015).

**Table 1:** **Non-associating engineered photoreceptors**.

**Class**	**Protein architecture^a^**	**Trade name**	**Cofactor**	**Size (aa)^b^**	**Activation/Deactivation λ_max_ (nm)**	**Dark recovery^c^**	**Optogenetic application**
LOV	*As*LOV2-*Ec*TrpR	LOV-TAP	FMN	240	450/dark	Seconds	DNA binding (Strickland et al., 2008)
	*As*LOV2 inserted into *Ec*DHFR	–	FMN	300	450/dark	Seconds	Enzymatic activity (Lee et al., 2008)
	*As*LOV2-*Hs*Rac1	PA-Rac1	FMN	320	450/dark	Seconds	GTPase signaling (Wu et al., 2009)
	*As*LOV2-*Hs*Stim1	LOVS1K	FMN	360	450/dark	Seconds	Ion channel activation (Pham et al., 2011)
	*As*LOV2-*Hs*Caspase 7	L57V	FMN	400	450/dark	Seconds	Caspase/apoptosis (Mills et al., 2012)
	*Da*αDTX-*As*LOV2	Lumitoxin	FMN	300	450/dark	Seconds	Ion channel inhibition (Schmidt et al., 2014)
	*At*LOV2-*Mm*Odc1 peptide	PSD	FMN	160	450/dark	Seconds	Protein degradation (Renicke et al., 2013)
	*As*LOV2-peptide	B-LID	FMN	150	450/dark	Seconds	Protein degradation (Bonger et al., 2014)
	*As*LOV2-*Hs*PKI	PA-PKI	FMN	160	450/dark	Seconds	Inhibition of endogenous Ser/Thr kinases (Yi et al., 2014)
	*As*LOV2-peptide	PA-MKI					
	*As*LOV2-peptides	LINuS	FMN	150	450/dark	Seconds	Nuclear transport (Niopek et al., 2014)
	*Bs*YtvA-*Bj*FixL	YF1, YHF	FMN	390	Dark/450	Minutes to hours	Prokaryotic gene expression (Möglich et al., 2009a, 2010a; Ohlendorf et al., 2012)
PYP	*Sc*GCN4-*Hh*PYP	–	pCA	160	450/dark	Seconds	DNA binding (Fan et al., 2011)
rhodopsin	*Bt*Rhodopsin-*Cg*β_2_AR	–	Retinal	350	500/dark	Seconds to minutes	cNMP signaling (Kim et al., 2005)
	*Bt*Rhodopsin-*Hs*α_1a_AR *Bt*Rhodopsin-*Cg*β_2_AR	OptoXR	Retinal	400 350	500 /dark	Seconds to minutes	cNMP and PLC signaling (Airan et al., 2009)
	*Mm*Melanopsin *Cr*Opsin	–	Retinal	500 330	480/light^d^ 500	Seconds Seconds	cNMP and PLC signaling (Karunarathne et al., 2013)
	*Mm*Melanopsin-*Mm*mGluR6	Opto-mGluR6	Retinal	500	480/light^d^	Seconds	vision restoration (Van Wyk et al., 2015)
	*Rr*Rhodopsin-*Hs*5-HT_1A_	Rh-CT_5-HT1A_	Retinal	350	485/dark	Seconds to minutes	Ion-channel activation (Oh et al., 2010)
bacterio-phytochrome	*Sp*Cph1-*Ec*EnvZ	Cph8	PCB	750	660/720	Minutes	Prokaryotic gene expression (Levskaya et al., 2005)
	*Dr*BPhy-*Hs*PDE2A	LAPD	BV	890	700/750	Minutes	cNMP hydrolysis (Gasser et al., 2014)
	*Rs*BPhG-*Ns*CyaB1	IlaC	BV	800	710/760	Minutes	cAMP formation (Ryu et al., 2014)

a *Species abbreviations as follows: As, Avena sativa; At, Arabidopsis thaliana; Bj, Bradyrhizobium japonicum; Bs, Bacillus subtilis; Bt, Bos taurus; Cg, Cricetulus griseus; Cr, Carybdea rastonii; Dr, Deinococcus radiodurans; Da, Dendroaspis angusticeps; Ec, Escherichia coli; Hh, Halorhodospira halophila; Hs, Homo sapiens; Mm, Mus musculus; Ns, Nostoc sp.; Rr, Rattus rattus; Rs, Rhodobacter sphaeroides; Sc, Saccharomyces cerevisiae; Sp, Synechocystis PCC6803; “peptide” refers to a short, synthetic sequence*.

b *Sizes are approximate*.

c *Time scales refer to intrinsic dark-recovery reaction; note that this is temperature-dependent and may be followed by slow biological processes*.

d *Melanopsin has been reported to be photochromic but the spectrum for deactivation has not been thoroughly determined (Ernst et al., 2014)*.

### Non-associating photoreceptors and optogenetic applications

This section treats non-associating photoreceptors where formation of the signaling state is not accompanied by changes in oligomeric state. Within this diverse class, linkers are either non-existent, in particular where sensor and effector modules are integrated into a single protein domain, as in rhodopsins, or they are of defined structure, usually of α-helical conformation. Due to their rigid structure, α helices and α-helical coiled coils are well-suited for transmitting conformational and dynamic changes over long distances between spatially distant sensors and effectors (Wolgemuth and Sun, 2006). Indeed, many natural signal receptors display sequence signatures indicative of helical and coiled-coil linkers (Anantharaman et al., 2006; Möglich et al., 2009a,b, 2010a; Rockwell et al., 2013). Furthermore, since α helices are self-contained structural elements stabilized by short-range contacts, locally confined order-disorder transitions, i.e., unfolding of helices, are enabled. Despite their disparity, non-associating photoreceptors can be further divided into two subcategories based on whether they chiefly capitalize on local unfolding (Section Light-regulated Order-disorder Transitions) or use other allosteric mechanisms involving tertiary and quaternary (apart from association) structural transitions (Section Light-regulated Tertiary and Quaternary Structural Transitions).

#### Light-regulated tertiary and quaternary structural transitions

The first subcategory of non-associating photoreceptors comprises a diverse group in which photoreception is not (primarily) accomplished by order-disorder transitions but by other tertiary and quaternary structural transitions, many of which are not yet understood in full molecular detail. Tracing back to seminal work by the Khorana group (Kim et al., 2005), the engineering of animal (type II) rhodopsin photoreceptors has been particularly successful (Airan et al., 2009; Karunarathne et al., 2013; Van Wyk et al., 2015). Type-II rhodopsins belong to the superfamily of G-protein-coupled receptors (GPCR) and consist of a retinal-binding seven-helix transmembrane photosensor module and an effector module formed by several intracellular loops; that is, both modules are integrated into a single protein domain (Ernst et al., 2014; Terakita and Nagata, 2014). Downstream signaling is mediated by heterotrimeric G proteins which specifically interact with the effector loops in signal-dependent manner. By exchanging these intracellular effector loops between type-II rhodopsins and other GPCRs, cellular signaling pathways can be rewired and put under light control. Following the initial demonstration (Kim et al., 2005), this engineering concept has later been popularized as “optoXR” (Airan et al., 2009). Replacement of the effector loops in bovine visual rhodopsin that originally mediate interactions with transducin (*G*_t_) by corresponding loops of the α_1_- and β_2_-adrenergic receptors yielded chimeric rhodopsins that achieve light control over *G*_s_- and *G*_q_-coupled signal pathways, i.e., control over cAMP (3′,5′-cyclic adenine monophosphate) production and phospholipase-C activity (Kim et al., 2005; Airan et al., 2009). However, a big drawback of this approach is the requirement for enzymatic regeneration of the 11-*cis* retinal chromophore of bovine rhodopsin once it underwent photoiosmerization to its all-*trans* form. This bottleneck can be overcome by resorting to rhodopsins that undergo reversible photoisomerization, as, e.g., melanopsin; in this way, *G*_i_, *G*_q_, and *G*_s_ signaling pathways have been put under light control (Karunarathne et al., 2013). In a very recent application of the optoXR concept, the activity of the metabotropic glutamate receptor mGluR6 could be controlled by light, thus yielding a promising tool for vision restoration (Van Wyk et al., 2015). Certain rhodopsins intrinsically mediate the desired type of G protein signaling and could hence immediately be used as optogenetic tools. As a prerequisite, correct expression and trafficking in heterologous hosts need to be ensured, for example by appending C-terminal localization signals to otherwise intact rhodopsins (Oh et al., 2010; Spoida et al., 2014).

For several homodimeric photoreceptors, engineering is based on the exchange of light-inert chemosensors for structurally and functionally homologous LOV (Möglich et al., 2009a, 2010a) or bacteriophytochrome (Levskaya et al., 2005; Gasser et al., 2014; Ryu et al., 2014) photosensors. For example, we replaced the oxygen-sensitive PAS B domain of the histidine kinase FixL from *Bradyrhizobium japonicum* with the LOV domain of *Bacillus subtilis* YtvA to obtain the photoreceptor YF1. Net histidine kinase activity of YF1 is repressed by more than 1000-fold under blue light (Möglich et al., 2009a) which underpins two systems for light-regulated gene expression in prokaryotes (Ohlendorf et al., 2012). YF1 variants differing in the linker connecting the LOV photosensor and histidine kinase effector modules displayed a striking heptad periodicity of activity and light regulation on linker length. Based on these observations, we posited (Möglich et al., 2009a) that these modules of the homodimeric photoreceptor are connected by a parallel α-helical coiled coil which has been borne out in the full-length crystal structure of YF1 (Diensthuber et al., 2013). Signals originating in the LOV photosensor could be transmitted to the distal effector via torque motions (supertwisting) of the coiled coil. In a derivative photoreceptor, denoted YHF, in which the PAS B domain is retained and combined with the *Bs*YtvA LOV domain, net kinase activity is regulated by oxygen and blue light in a positive cooperative manner (Möglich et al., 2010a). A conceptually similar, prior study yielded the red-light-repressed photoreceptor Cph8 which consists of a cyanobacterial phytochrome photosenor module, that comprises PAS, GAF, and PHY domains and uses a phycocyanobilin chromophore, and the *E. coli* histidine kinase EnvZ (Levskaya et al., 2005). Recently, bacteriophytochrome photosensors have also been used in the engineering of red-light-activated, far-red-light-reversible actuators of cyclic-nucleotide metabolism (Gasser et al., 2014; Ryu et al., 2014). For one, we substituted the two GAF sensor domains of the human phosphodiesterase 2A for the biliverdin-binding PAS-GAF-PHY tandem of *Deinococcus radiodurans* bacteriophytochrome to engineer the photoreceptor LAPD (Gasser et al., 2014). Hydrolysis of cAMP and cGMP (3′,5′-cyclic guanosine monophosphate) by LAPD could be enhanced by up to seven-fold by red light and could be attenuated by far-red light. Studies in cell culture and zebrafish confirmed *in vivo* functionality and revealed that biliverdin is sufficiently available endogenously and hence needs not be added exogenously. The implementation of a closely related design concept yielded the red-light-regulated adenylate cyclase IlaC that recombines the PAS-GAF-PHY photosensory module of the *Rhodobacter sphaeroides* bacteriophytochrome BphG1 with the catalytic domain of *Nostoc* sp. CyaB1 (Ryu et al., 2014). The cyclase activity of IlaC could be enhanced by around six-fold under red light, and functionality in nematodes was demonstrated. For both LAPD and IlaC, a strong dependence of catalytic activity and regulation on the length of the linker between photosensor and effector modules has been observed. Although these dependencies were less pronounced than for YF1, it is nonetheless conceivable that similar mechanisms for signal propagation are at play in YF1, LAPD, and IlaC. While the verdict is still out, future bacteriophytochrome engineering efforts will doubtless benefit from a ground-breaking recent study which revealed that the isolated PAS-GAF-PHY tandem of *D. radiodurans* bacteriophytochrome undergoes a pivot motion upon red-light absorption (Figure 3C) (Takala et al., 2014); recent findings imply a similar mechanism in plant phytochromes, too (Burgie et al., 2014; Burgie and Vierstra, 2014).

#### Light-regulated order-disorder transitions

Within the second subcategory of non-associating photoreceptors, several studies have exploited the light-regulated unfolding of the C-terminal Jα helix of phototropin LOV domains, chiefly the *As*LOV2 domain (Harper et al., 2003), in one of essentially three ways: mutually exclusive folding (Strickland et al., 2008), steric restriction of effector active sites (Lee et al., 2008; Wu et al., 2009; Pham et al., 2011; Mills et al., 2012; Schmidt et al., 2014), or uncaging of peptide epitopes (Lungu et al., 2012; Strickland et al., 2012; Renicke et al., 2013; Bonger et al., 2014; Niopek et al., 2014; Yi et al., 2014; Guntas et al., 2015). As explained in Section Thermodynamics of Photoreceptors, in the dark the Jα helix is predominantly folded and docked against the LOV core domain, but upon blue-light absorption it predominantly dissociates and unfolds. In the LOV-TAP protein (Strickland et al., 2008), the C-terminal Jα helix of *As*LOV2 is fused with an N-terminal helix of the *E. coli* TrpR repressor such that steric interference between the two modules results in mutually exclusive folding: the joint helix can either fold unto the *As*LOV2 or the TrpR domain but not unto both simultaneously; correct folding and DNA affinity of TrpR could thus be modestly regulated by blue light.

The *As*LOV2 domain has also been inserted into surface loops of an effector domain to put biological activity under light control, albeit yielding poor overall activity and dynamic range for light regulation in the initial implementation (Lee et al., 2008). However, the successful design of several light-inert receptors by domain insertion (Makhlynets et al., 2015; Stein and Alexandrov, 2015) strongly suggests that the basic concept is viable and suitable for the regulation of diverse effectors. In a related strategy, the *As*LOV2 domain is connected via its Jα helix to selected effector domains such that their active site is occluded (Wu et al., 2009). Once Jα unfolds, the effector domain dissociates from *As*LOV2, steric restriction to the active site is relieved, and biological activity is enhanced. This concept was pioneered in the regulation of the small GTPase Rac1 which afforded blue-light control over cell motility (Wu et al., 2009). Crystallographic analysis revealed an adventitious structural contact in the resultant PA-Rac1 construct between the *As*LOV2 and Rac1 domains which was not rationally planned but is apparently necessary for light regulation. The requirement for such, highly specific interactions may be the reason why the successful design strategy could not readily be transferred to homologous GTPases, e.g., Cdc42 (Wu et al., 2009). Later on, the concept of steric restriction to active sites has also been applied to the regulation of Ca^2+^ ion channels (Pham et al., 2011) and caspases (Mills et al., 2012). In the related lumitoxin approach, peptide toxins are appended N-terminally to a membrane-tethered *As*LOV2 domain such that the toxins are sequestered from their inhibitory sites on endogenous potassium channels (Schmidt et al., 2014). Light-induced unfolding of the Jα helix grants the *As*LOV2 domain enhanced mobility and allows the associated toxin to reach and inhibit the ion channel.

In the particularly versatile “peptide uncaging” strategy, a peptide epitope is interleaved with or appended to the Jα helix such that its folding and solvent exposure become subject to light control. Once the peptide epitope is undocked from the LOV core domain following light absorption, it can trigger downstream signaling events. This principle has been implemented to achieve light-regulated protein degradation (Renicke et al., 2013; Bonger et al., 2014), inhibition of endogenous serine/threonine kinases (Yi et al., 2014), and nuclear transport (Niopek et al., 2014). Unfolding of peptide epitopes interleaved with Jα also forms the basis for several associating photoreceptors (cf. Section Associating Photoreceptors and Optogenetic Applications) (Lungu et al., 2012; Strickland et al., 2012; Guntas et al., 2015). Note that the incorporation of peptide epitopes may significantly alter the stabilities of the α-helical (T) and unfolded (R) states of Jα and thereby affect the equilibrium between these states (cf. Section Thermodynamics of Photoreceptors and Figures 2C–E). To compensate for such effects and to optimize dynamic range as dictated by application, several mutations within the *As*LOV2 core domain and the Jα helix can be deployed that deliberately (de)stabilize the α-helical vs. the unfolded states (Strickland et al., 2010, 2012; Lungu et al., 2012; Zayner et al., 2012; Guntas et al., 2015).

Notably, light-regulated unfolding is by no means exclusive to *As*LOV2 but also occurs in other photoreceptors; as a case in point, the light-induced unfolding of an N-terminal segment of the photoactive yellow protein (PYP) has been exploited in subjecting the DNA affinity of an N-terminally appended GCN4 leucine-zipper protein under light control (Fan et al., 2011).

### Associating photoreceptors and optogenetic applications

Since many biological processes involve protein oligomerization, often dimerization (Klemm et al., 1998), it should come as no surprise that (i) many sensory photoreceptors exist in Nature that undergo light-dependent association reactions; and (ii) that these photoreceptors have proven particularly powerful in optogenetic engineering. Associating photoreceptors can be further subdivided into homo- vs. heteroassociation, reactions of different stoichiometry (dimer, oligomer), and into light-triggered association vs. dissociation (cf. Section Thermodynamics of Photoreceptors). Regulation of biological activity in this class amounts to co-localization of interacting proteins and/or recruitment to subcellular compartments; notably, this concept extends to the control of split proteins, well established for chemically induced dimerization (Pollock and Clackson, 2002). For this engineering concept, photosensor and effector modules need to be physically connected but they generally need not directly interact with one another (in fact, it is often preferable, they do not). Constraints on linker identity are hence less demanding than for non-associating photoreceptors (cf. Section Non-associating Photoreceptors and Optogenetic Applications), and engineering ideally becomes less challenging and more predictive. Notably, the engineering of associating photoreceptors shares aspects with the labeling with fluorescent proteins (Tsien, 2009), in that linkers are often short, flexible and predominantly hydrophilic. Various associating photoreceptors with sensitivity to different portions of the visible electromagnetic spectrum (cf. Figure 1) have been used in engineering, and we provide an overview in Table 2 and in the following.

**Table 2:** **Associating engineered photoreceptors**.

**Photoreceptor (pair)^a^**	**Cofactor**	**Size (aa)**	**Association/Dissociation λ_max_ (nm)**	**Dark recovery**	**Optogenetic application**
*At*UVR8	–	440	Dark/280	Hours	Protein sceretion (Chen et al., 2013)
*At*UVR8: *At*COP1	–	440: 340	Dark/280	Hours	Eukaryotic gene expression (Crefcoeur et al., 2013; Müller et al., 2013b)
*Nc*VVD	FAD	150	450/dark	Hours	Eukaryotic gene expression (Wang et al., 2012; Nihongaki et al., 2014) caspase/apoptosis (Nihongaki et al., 2014)
*Nc*VVD I52R/M55R: *Nc*VVD I52D/M55G	FAD	150	450/dark	Seconds to hours	Recruitment, phosphoinositide metabolism (Kawano et al., 2015)
*Pt*Aureochrome [*Vf*Aureochrome]	FMN	150	450/dark	Minutes to hours	RTK signaling (Grusch et al., 2014)
*El*EL222	FMN	222	450/dark	Seconds	Eukaryotic gene expression (Motta-Mena et al., 2014)
*Rs*LOV	FMN	176	Dark/450	Minutes	Biophysical characterization (Conrad et al., 2013)
*At*FKF1: *At*GIGANTEA	FMN	619 (isolated LOV 160): 1173	450/dark	Hours—∞	Eukaryotic gene expression (Yazawa et al., 2009; Polstein and Gersbach, 2012) Ion-channel gating (Dixon et al., 2012)
*As*LOV2-peptide: *Hs*Erbin_PDZ	FMN	160: 194	450/dark	Seconds	MAP kinase signaling (Strickland et al., 2012) Motor proteins and intracellular transport (Van Bergeijk et al., 2015)
*As*LOV2-*Sf*IpaA: *Hs*Vinculin_D1	FMN	160: 260	450/dark	Seconds	Eukaryotic gene expression (Lungu et al., 2012)
*As*LOV2-*Ec*SsrA: *Ec*SspB	FMN	160: 165	450/dark	Seconds	Recruitment, GTPase signaling (Lungu et al., 2012; Guntas et al., 2015)
peptide-*Hh*PYP	pCA	150	Dark/450	Seconds	Biophysical characterization (Reis et al., 2014)
*At*Cry2	FAD (MTHF)	498	450/dark	Minutes	Clustering, GTPase signaling, eukaryotic gene expression (Bugaj et al., 2013) RTK signaling (Chang et al., 2014; Kim et al., 2014) MAP kinase signaling (Wend et al., 2014) Sequestration-based protein inhibition (Lee et al., 2014) Clustering, clathrin-mediated endocytosis, SH3 signaling (Taslimi et al., 2014)
*At*Cry2: *At*CIB1	FAD (MTHF)	498: 170	450/dark	Minutes	Recruitment, eukaryotic gene expression, recombination (Kennedy et al., 2010) Eukaryotic gene expression (Liu et al., 2012) Phosphoinositide metabolism (Idevall-Hagren et al., 2012; Kakumoto and Nakata, 2013) MAP kinase signaling (Aoki et al., 2013; Zhang et al., 2014) Sequestration-based protein inhibition (Lee et al., 2014) Expression of endogenous eukaryotic genes (Konermann et al., 2013; Nihongaki et al., 2015; Polstein and Gersbach, 2015)
*Pe*Dronpa K145N	–	257	400/500	Hours to days	GTPase signaling, protease (Zhou et al., 2012)
*At*PhyA/*At*PhyB: *At*PIF3/*At*PIF6	PΦB or PCB	620, 910: 100, 520	650/750	Minutes	Eukaryotic gene expression (Shimizu-Sato et al., 2002; Müller et al., 2013b) Protein splicing (Tyszkiewicz and Muir, 2008) GTPase signaling (Levskaya et al., 2009) MAP kinase signaling (Toettcher et al., 2013) Nuclear transport (Beyer et al., 2015a) Recruitment to intracellular compartments (Yang et al., 2013)
*Sp*Cph1 PAS-GAF-PHY	PCB	510	660/720	Minutes	Light-induced homodimerization (Strauss et al., 2005)

a *Species abbreviations cf. Table 1 and as follows: El, Erythrobacter litoralis; Nc, Neurospora crassa; Pe, Pectiniidae cDNA; Pt, Phaeodactylum tricornutum; Rs, Rhodobacter sphaeroides; Sf, Shigella flexneri; Vf, Vaucheria frigida*.

The plant photoreceptor UVR8 (Brown et al., 2005) does not require any cofactors but utilizes tryptophan chromophores to sense UV-B light. Photon absorption induces disruption of the homodimeric UVR8 receptor and enables the constituent monomers to associate with COP1 (Christie et al., 2012; Wu et al., 2012). In the absence of additional plant proteins, the dark-recovery reaction of UVR8 is exceedingly slow (hours to days), thus rendering pertinent optogenetic applications effectively irreversible on most biologically relevant time scales. The dissociation reaction of UVR8 has been exploited in subjecting protein secretion under UV-light control (Chen et al., 2013), and the interaction with COP1 provides the basis for two closely similar systems for UV-light-regulated expression of transgenes in eukaryotic cells (Crefcoeur et al., 2013; Müller et al., 2013b).

Several optogenetic applications resort to associating photoreceptors of the flavin-binding light-oxygen-voltage (LOV) family (Conrad et al., 2014). Certain LOV domains, e.g., those of *Neurospora crassa* Vivid (*Nc*VVD, Lamb et al., 2008, Figure 3B), stramenopile aureochromes (Takahashi et al., 2007) and *Erythrobacter litoralis* EL222 (Nash et al., 2011), assemble into homodimers upon blue-light absorption. While mechanistic details often remain to be elucidated, in all cases flanking, mostly α-helical extensions N- and C-terminal to the LOV core domain (Möglich et al., 2009b; Conrad et al., 2014) apparently play key roles in mediating the association reactions; mutations that (de)stabilize these regions, e.g., I52C (Nihongaki et al., 2014) and N56K (Wang et al., 2012), C71V (Zoltowski and Crane, 2008) in *Nc*VVD, can be applied for optimization of the dynamic range of the photoresponse. The *Nc*VVD LOV domain provides the basis for both the “LightON” system for light-induced expression of transgenes in eukaryotic hosts (Wang et al., 2012; Nihongaki et al., 2014) and for the engineering of a light-inducible caspase that elicits apoptosis (Nihongaki et al., 2014). Aureochrome proteins, originally discovered in the stramenopile alga *Vaucheria frigida* (Takahashi et al., 2007), are basic-zipper transcription factors that harbor a LOV domain at their C terminus, thus diverging from the more common topology of N-terminally situated LOV photosensors (Möglich et al., 2010b). By appending aureochrome LOV domains to the intracellular C terminus of receptor tyrosine kinases (RTK), these RTKs could be dimerized upon blue-light exposure, and MAP kinase signaling could be triggered (Grusch et al., 2014). Notably, the natural C-terminal topology of aureochrome LOV domains had to be recapitulated in the engineered RTKs to achieve efficient light regulation; by contrast, use of other associating LOV domains with N-terminal topology failed to yield light-regulated RTKs. EL222 is a bacterial LOV transcription factor that undergoes dimerization and DNA binding to cognate promoters under blue light (Nash et al., 2011). By connecting eukaryotic transactivation domains to EL222, a system for blue-light-induced transgene expression in eukaryotic cell culture and zebrafish larvae was furnished (Motta-Mena et al., 2014).

Although not yet used in photoreceptor engineering, a LOV protein from *Rhodobacter sphaeroides* (*Rs*LOV, Conrad et al., 2013) should be an attractive building block as this homodimeric photoreceptor dissociates into monomers upon blue-light absorption, i.e., it displays the opposite signal polarity than the above LOV systems. A functionally similar system has been engineered on the basis of a circularly permuted PYP protein; a domain-swapped dimer of this modified PYP dissociates into monomers under blue light (Reis et al., 2014).

Several LOV-based systems are available that mediate light-regulated heteroassociation. The plant FKF1 LOV protein associates with the GIGANTEA (GI) protein in blue-light-stimulated manner, and the FKF1:GI pair has been used in the engineering of systems for light regulation of transgene expression in eukaryotic hosts (Yazawa et al., 2009; Polstein and Gersbach, 2012) and ion-channel gating (Dixon et al., 2012). However, wider adoption has been hampered by the large size of GI and the very slow dark recovery of FKF1, lasting several hours to days. Given these shortcomings, several engineered systems for light-regulated heteroassociation based on the *As*LOV2 domain fill a niche not yet fully covered by naturally occurring, associating photoreceptors (Lungu et al., 2012; Strickland et al., 2012). These systems capitalize on the light-induced unfolding of the C-terminal Jα helix of *As*LOV2 and interleaved or appended peptide eptiopes (cf. Section Light-regulated Order-disorder Transitions). Once the peptide epitope is liberated upon light absorption, it can enter intermolecular interactions with a specific binding protein. Ideally, both the modified *As*LOV2 domain and the cognate binding protein are small in size and usable in different modular contexts, e.g., appended N- or C-terminally to target proteins. The TULIP system uses a modified PDZ domain as the binding protein and has been applied to subject MAP kinase signaling (Strickland et al., 2012) and intracellular transport under light control (Van Bergeijk et al., 2015). Two other systems rely on interactions of peptide epitopes with the vinculin and SsrB proteins, respectively (Lungu et al., 2012); a recently improved version of the SsrB-based system appears to outperform both the TULIP and the vinculin-based systems and has been applied to the regulation of GTPase signaling (Guntas et al., 2015). Very recently, the *Nc*VVD photoreceptor, which naturally assembles into a homodimer upon blue-light absorption (cf. above), has been re-engineered into a system for blue-light-induced heterodimerization (Kawano et al., 2015).

The currently most commonly applied photoreceptor systems for light-activated association reactions are based on *A. thaliana* cryptochrome 2 (*At*Cry2) which binds a FAD chromophore and a MTHF (methenyltetrahydrofolate) antenna pigment to respond to blue light (Conrad et al., 2014). Motivated by the initial demonstration that the blue-light-induced dimerization of *At*Cry2 with its partner protein *At*CIB1 (Liu et al., 2008) can be functionally reconstituted in heterologous hosts (Kennedy et al., 2010), the *At*Cry2:*At*CIB1 pair has been used for light control of various biological processes, including expression of transgenes (Kennedy et al., 2010; Liu et al., 2012) and endogenous genes (Konermann et al., 2013; Nihongaki et al., 2015; Polstein and Gersbach, 2015) in eukaryotic cells, DNA recombination (Kennedy et al., 2010), phosphoinositide metabolism (Idevall-Hagren et al., 2012; Kakumoto and Nakata, 2013) and MAP kinase signaling (Aoki et al., 2013; Zhang et al., 2014). A creative and potentially versatile approach is provided by the LARIAT strategy which combines the *At*Cry2:*At*CIB1 pair with multivalent adapter proteins. In this way, diverse target proteins can be recruited in light-triggered manner to larger protein agglomerates with commensurate inhibition of their biological activity (Lee et al., 2014). Studies by Tucker and coworkers showed that N-terminally truncated versions of both *At*Cry2 (residues 1–498) and *At*CIB1 (1–170) suffice for mediating light-induced heteroassociation (Kennedy et al., 2010).

*At*Cry2 is known to reversibly assemble into oligomers under blue light, as for example evident as punctae formation in plant cell nuclei (Yu et al., 2009). Light-dependent clustering of *At*Cry2 can be reconstituted in heterologous hosts and has been exploited in the regulation of GTPase signaling (Bugaj et al., 2013), expression of eukaryotic transgenes (Bugaj et al., 2013), MAP kinase signaling (Wend et al., 2014), RTK signaling (Chang et al., 2014; Kim et al., 2014), SH3 signaling (Taslimi et al., 2014) and clathrin-mediated endocytosis (Taslimi et al., 2014). As discussed in Section Thermodynamics of Photoreceptors, higher-order oligomerization reactions strongly depend on the total monomer concentration: if concentrations are too low, light-induced clustering will be inefficient; if concentrations are too high, clusters will exist under both dark and light conditions. Moreover, the efficiency of *At*Cry2 cluster formation strongly depends upon applied light dose and cell type under investigation (Taslimi et al., 2014). Recently, the E490G variant of *At*Cry2, denoted “Cry2olig,” has been reported as having increased propensity for light-induced oligomerization (Taslimi et al., 2014). While the great utility of Cry2olig is beyond doubt, it is not clear whether improved clustering is entirely due to a higher affinity (*K*^R^_D_), or there is a contribution of enhanced expression (cf. Section Thermodynamics of Photoreceptors).

The photoswitchable, fluorescent protein Dronpa has also been converted into a photoreceptor for optogenetics (Zhou et al., 2012). While the K145N mutant of Dronpa undergoes a weak homotetramerization reaction, it can also form a heterodimer with the wild-type protein. In both cases, association is triggered by violet light (400 nm) and can actively be reversed by cyan light (500 nm). Using these Dronpa variants, GTPase signaling and protease activity could be controlled by light. Of key advantage, the chromophore of Dronpa is formed autocatalytically from three residues, and thus no cofactor is required.

At the red end of the visible spectrum, the *A. thaliana* phytochrome *At*PhyB and its interacting proteins *At*PIF3 and *At*PIF6 provide commonly used systems for light-regulated heteroassociation reactions. Note that in the initial implementation of the Phy:PIF system *At*PhyA was used (Shimizu-Sato et al., 2002), but later applications instead employed *At*PhyB. A key advantage of *At*Phys is their photochromic nature (cf. Section Photochromic Photoreceptors): red light (of around 650 nm) promotes association, far-red light (above 700 nm) promotes dissociation. However, plant Phys require either their natural cofactor phytochromobilin (PΦB) or the cyanobacterial phycocyanobilin (PCB) as a surrogate, neither of which occur naturally in most tissues that are targeted by optogenetics. As a consequence, chromophore has to be added exogenously, or additional genes for endogenous production of PCB have to be introduced to target tissues as well (Müller et al., 2013c). Despite this limitation, *At*Phy:*At*PIF-based systems have been successfully used for regulating by light gene expression of eukaryotic transgenes (Shimizu-Sato et al., 2002; Müller et al., 2013b), protein splicing (Tyszkiewicz and Muir, 2008), GTPase signaling (Levskaya et al., 2009), MAP kinase signaling (Toettcher et al., 2013), nuclear transport (Beyer et al., 2015a), as well as sequestration to subcellular compartments (Yang et al., 2013). Similar to the cryptochrome case above, N-terminal fragments of *At*PhyA/B (either residues 1–650 or 1–910) and *At*PIF3/6 (residues 1–100) suffice to elicit red-light-activated, far-red-light-reversible heteroassociation (Levskaya et al., 2009; Müller et al., 2013a,b), with the caveat that a recent study implies that in a yeast transcriptional assay full-length PIF3 supports a higher dynamic range of light activation than the N-terminally truncated version (Pathak et al., 2014). Lastly, the PAS-GAF-PHY tandem of the cyanobacterial phytochrome Cph1 has been shown to associate into a homodimer upon red-light exposure which could be capitalized on in photoreceptor engineering (Strauss et al., 2005).

In summary, a variety of photoreceptor systems exist that achieve different light sensitivity and stoichiometries for association/dissociation reactions. Based on the number of successful engineering examples, the *At*Cry2:*At*CIB1 system currently appears to have the edge; key benefits of this system are low dark activity, reasonably compact tag size, use of widely occurring chromophores, and excitability with widespread illumination sources. By contrast, the *At*Phy:*At*PIF systems suffer from larger tag size, dependence on exogenous chromophore addition (or endogenous heterologous production, Müller et al., 2013c), and requirement for less common light sources. However, the photochromic nature of the Phy:PIF system and the very high dynamic range in at least some applications (Shimizu-Sato et al., 2002) certainly speak strongly in favor of this system. A palpable dearth of side-by-side comparisons between systems has recently been addressed in a laudable study that investigates the performance of several associating photoreceptors under comparable test conditions (Pathak et al., 2014). At least under the specific assay settings tested, *At*PhyB:*At*PIF3 displayed a higher dynamic range for light-regulated gene expression in yeast than *At*Cry2:*At*CIB1 did. While not included in the performance comparison, the recently improved LOV-SsrB system appears an attractive option for light-regulated heteroassociation (Guntas et al., 2015). However, we caution that the performance of associating photoreceptor systems apparently depends on the context in which they are used and tested (Pathak et al., 2014). As a consequence, for some applications a given associating photoreceptor may be the best choice, while in other applications it may be an altogether different one.

## Photochromic photoreceptors

**Figure 5:**
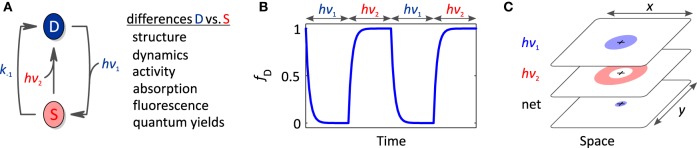
**Photochromic photoreceptors**. **(A)** Photochromic photoreceptors derive added versatility from the ability to actively and bidirectionally interconvert dark-adapted and signaling states via absorption of photons of the activating (*h*ν_1_) and deactivating wavelengths (*h*ν_2_). As these states generally differ in their structure, dynamics, biological activity and spectral properties, light can be used to reversibly toggle these properties. **(B)** Light-driven, bidirectional interconversion enables better time resolution than for non-photochromic photoreceptors. **(C)** Likewise, superior local control is achievable by spatially interleaving different light colors. A setup akin to STED super-resolution microscopy might be implemented to achieve photoreceptor stimulation volumes with supreme spatial resolution.

Bacterial and plant phytochromes, cyanobacteriochromes and many rhodopsins are so-called photochromic photoreceptors (Table 3) in which the metastable signaling state S, formed after absorption of one photon, can be actively converted back to the dark-adapted state D by absorption of another photon (mostly of different color) (Figure 5A). To discriminate between these two light-driven reactions, in the following we denote them as activating and deactivating processes, respectively.

**Table 3:** **Photochromic photoreceptors**.

**Class**	**Subclass/Family**	**Representative/Paradigm^a^**	**Cofactor**	**Activation λ_max_ (nm)**	**Deactivation λ_max_ (nm)**	**References**
Rhodopsin	Type-I (microbial)	*Cr*HKR1	All-*trans*↔13-*cis* retinal	380	400	Luck et al., 2012
		*Cr*ChR2 C128A	All-*trans*↔13-*cis* retinal	470	530	Berndt et al., 2009
	Type-II (animal)	*Mm*Melanopsin	11-*cis*↔all-*trans* retinal	465	475	Freedman et al., 1999
		*Lj*Parapinopsin	11-*cis*↔all-*trans* retinal	370	515	Koyanagi et al., 2004
Phytochrome	Plant	*At*PhyB	PΦB orPCB	650–670	700–730	Rockwell et al., 2006; Rockwell and Lagarias, 2010
	Bacterial and fungal	*Dr*BphP	BV	700	750	Hughes et al., 1997; Rockwell et al., 2006; Rockwell and Lagarias, 2010
	Cyanobacterial	*Sy*Cph1	PCB	660	705	Rockwell et al., 2006; Rockwell and Lagarias, 2010
	Algal	*Dt*Phy1	PCB	595	725	Rockwell et al., 2014
		*Cp*GPS1	PCB	440	635	Rockwell et al., 2014
		*Es*PHL1	PϕB	690	565	Rockwell et al., 2014
Cyanobacteriochrome	Green/red CBCRs	*Sy*CcaS	PCB	535	670	Hirose et al., 2008
	Red/green CBCRs	*An*PixJ *Am*AM1_1557	PCB PCB (or BV)	650 650 (700)	545 545 (620)	Ikeuchi and Ishizuka, 2008; Rockwell and Lagarias, 2010; Narikawa et al., 2015
	DXCF CBCRs	*Te*PixJ *Np*F4973	PCB→PVB PCB	430 435	530 600	Ikeuchi and Ishizuka, 2008; Rockwell and Lagarias, 2010
	Insert-Cys CBCRs	*Np*F2164	PCB→PVB	400	590	Ikeuchi and Ishizuka, 2008; Rockwell and Lagarias, 2010
	Novel dual-Cys CBCR	*Am*AM1_1186	PCB	640	415	Ikeuchi and Ishizuka, 2008; Rockwell and Lagarias, 2010
Other	LOV	*Bs*YtvA	FMN	450	355	Losi et al., 2013
	Cryptochrome	*Cr*Cry	FAD	450	635	Beel et al., 2012
	Dronpa	*Pe*Dronpa	–	400	500	Zhou et al., 2012

a *Species abbreviations cf. Tables 1, 2 and as follows: Am, Acaryochloris marina; An, Anabaena sp.; Cp, Cyanophora paradoxa; Cr, Chlamydomonas reinhardtii; Dt, Dolichomastix tenuilepis; Es, Ectocarpus siliculosus; Lj, Lethenteron japonica; Np, Nostoc punctiforme; Te, Thermosynechococcus elongates*.

### Classes of photochromic photoreceptors

Rhodopsins divide into microbial-type proteins (type I), which are exemplified by bacteriorhodopsin, and animal-type proteins (type II), which form a subclass of GPCRs (Ernst et al., 2014). Whereas in microbial rhodopsins the retinal chromophore undergoes an all-*trans* to 13-*cis* isomerization upon light absorption, in animal rhodopsins the retinal isomerizes from 11-*cis* to all-*trans* or *vice versa* (Ernst et al., 2014; Terakita and Nagata, 2014). In vertebrate visual opsins the chromophore is consumed after one isomerization event and the all-*trans* photoproduct needs to be recycled enzymatically to the 11-*cis* educt. By contrast, most microbial and non-visual animal rhodopsins are bistable, i.e., the signaling state thermally reverts to the dark-adapted state or it can be actively reverted by light absorption (Terakita and Nagata, 2014). In some rhodopsins, e.g., in channelrhodopsins (Berndt et al., 2009), mutations have been identified that greatly stabilize photocycle intermediates. As detailed above, originally bovine visual rhodopsins have been employed in the engineering of light-regulated GPCRs, but the design principle (Kim et al., 2005) also extends to other animal rhodopsins which offer bistable switching, e.g., vertebrate melanopsin (Freedman et al., 1999) or certain catfish and lamprey rhodopsins (Blackshaw and Snyder, 1997; Koyanagi et al., 2004). In addition, rhodopsin photosensors covalently linked to enzymatic effectors are of interest: the so-called HKR1 rhodopsin from *C. reinhardtii* bears a histidine-kinase effector and can be bidirectionally switched by UV-A and blue light, respectively (Luck et al., 2012); furthermore, a rhodopsin photosensor connected to a guanylate-cyclase effector was recently reported (Avelar et al., 2014).

Phytochromes and cyanobacteriochromes all share a canonical photochemical mechanism in which the primary event is *Z*/*E* isomerization around the C15=C16 double bond of a linear tetrapyrrole (bilin) chromophore covalently attached to a conserved cysteine residue (Rockwell et al., 2006; Rockwell and Lagarias, 2010). As both the 15*Z*→15*E* and the 15*E*→15*Z* isomerization reactions can be driven by light, photochromicity is an inherent property of all bilin-based photoreceptors. The 15*Z* form commonly absorbs light at somewhat shorter wavelengths than the 15*E* form, but there are notable exceptions among the CBCRs (Ikeuchi and Ishizuka, 2008; Rockwell and Lagarias, 2010). Plant and cyanobacterial phytochromes employ the reduced bilin chromophores phycocyanobilin (PCB) and phytochromobilin (PΦB) and transition between red-light-absorbing 15*Z* (λ_max_ ≈ 650–670 nm) and far-red-light-absorbing 15*E* states (λ_max_ ≈ 700–730 nm). Bacterial phytochromes instead use the more oxidized biliverdin which gives rise to a red shift of about 30 nm for both states (Rockwell et al., 2006). In conventional Phys the 15*Z* state is the thermally most stable form predominating in the dark; by contrast, in the so-called bathyphytochromes, the 15*E* state is more stable (Rockwell et al., 2006; Rockwell and Lagarias, 2010). In an unexpected twist, certain marine alga were recently found to possess phytochromes with absorption maxima for their 15*Z* and 15*E* states that are not in the red/far-red region but essentially cover the entire visible spectrum (Rockwell et al., 2014) and that thus rival cyanobacteriochromes in their spectral diversity. Whereas the photosensor of canonical Phys is composed of PAS, GAF and PHY domains, CBCR photosensors simply consist of a sole GAF domain and thereby offer a much smaller footprint (Ikeuchi and Ishizuka, 2008; Rockwell and Lagarias, 2010). CBCRs distribute into at least four distinct categories, namely red/green, green/red, DXCF and insert-Cys types; as a group, CBCRs display a bewildering spectral and mechanistic diversity and cover the entire UV-A/B and visible spectrum (Ikeuchi and Ishizuka, 2008; Rockwell and Lagarias, 2010). The photocycle of red/green and green/red CBCRs resembles that of traditional red/far-red-responsive plant phytochromes, with 15*Z*↔15*E* isomerization around the C15=C16 double bond of the chromophore as the principal event (Ikeuchi and Ishizuka, 2008; Rockwell and Lagarias, 2010). By contrast, in the DXCF and insert-Cys classes which together form the dual-cysteine CBCRs, the 15*Z*/15*E* isomerization is accompanied by the reversible formation of a covalent thioether bond between a second conserved cysteine and atom C10 of the bilin (Rockwell et al., 2011; Burgie et al., 2013; Narikawa et al., 2013) (Figure 3D). Note that in certain DXCF CBCRs the PCB chromophore is autocatalytically isomerized to phycoviolobilin (PVB) which absorbs at shorter wavelengths than PCB (Ishizuka et al., 2011). Intriguingly, the CBCR family may even be more diverse than the above four categories capture: a recently discovered dual-cysteine CBCR fits in neither the DXCF or insert-Cys categories and displays the largest spectral shift observed in CBCRs to date, between a red-absorbing 15*Z* state and a blue-absorbing 15*E* state (Narikawa et al., 2014). Cyanobacteriochromes commonly use the reduced bilins phycocyanobilin and phycoviolobilin as chromophores which generally do not occur in most organisms and cells targeted by optogenetics. However, a recent report (Narikawa et al., 2015) describes a CBCR that binds biliverdin, which widely occurs in many tissues (Shu et al., 2009; Filonov et al., 2011; Gasser et al., 2014), with similar affinity as phycocyanobilin. If biliverdin binding can be extended to other CBCRs and plant Phys, optogenetic deployment of these photoreceptors would be greatly facilitated.

Beyond rhodopsins and the bilin-dependent families, several other photoreceptors also offer photochromic, bidirectional switching. As discussed above, the fluorescent protein Dronpa has been configured into a sensory photoreceptor while retaining its spectral properties including bidirectional switching by violet and cyan light, respectively (Zhou et al., 2012). Furthermore, the signaling state in plant cryptochromes is formed via photoreduction of oxidized FAD to the partially reduced, neutral semiquinone radical form FADH• (Conrad et al., 2014); as shown for a cryptochrome from *C. reinhardtii* (Beel et al., 2012), absorption of a second photon of longer wavelength can drive full reduction to the hydroquinone state FADH_2_ with concomitant impact on downstream signaling. Finally, in LOV proteins the blue-light-induced adduct between a flavin-nucleotide chromophore and a cysteine residue can be disrupted by UV light to regenerate the dark-adapted state, albeit with low quantum yield (Losi et al., 2013).

### Properties of photochromic photoreceptors

Owing to bidirectional and reversible switching between the dark-adapted and signaling states, photochromic photoreceptors afford a number of unique advantages. By applying defined mixtures of the activating and deactivating wavelengths, precise ratios of the states D and S can be set and maintained over long times (Toettcher et al., 2013). Furthermore, the bidirectional photoswitching allows the precise confining of the signaling state in both time and space, thus potentially offering higher spatiotemporal resolution than otherwise feasible (Figures 5B,C). As detailed in Section Photoreceptor Fundamentals (also cf. Figure 2A), *off*-kinetics of light responses are strongly determined by the rate constant for dark recovery *k*_−1_. As one remedy, mutations can be introduced that accelerate the dark recovery; however, such mutations concomitantly increase the light intensities required at photostationary conditions for significant population of the signaling state (cf. Section Photoreceptor Fundamentals). A direct, active means of depleting the signaling state is afforded in photochromic photoreceptors via illumination with the “deactivating” wavelength. Formation of the signaling state and downstream signaling events can hence be controlled with superior temporal precision (Figure 5B). If activating and deactivating wavelengths are interleaved in space rather than time, enhanced spatial resolution of formation of the signaling state and elicited physiological responses may be obtained (Figure 5C). The ultimate in spatial resolution can arguably be achieved by implementing an illumination scheme corresponding to that underpinning stimulated-emission-depletion (STED) super-resolution microscopy (Hell and Wichmann, 1994), which banks on the fact that individual light waves are subject to the diffraction limit but the spatial superposition of several light waves need not be. Briefly, a spherical illumination profile of the activating wavelength could be superposed with a dough-nut-shaped illumination profile of the deactivating wavelength. In the spatial region where these profiles overlap, a photochromic photoreceptor would be continuously toggled between the dark-adapted and signaling states. The net result of this tug-of-war would be determined by the intensities of activating and deactivating wavelengths as a function of space as well as the quantum yields for the activating and deactivating reactions. Whereas in STED the deactivating wavelength promotes an emissive transition originating from a short-lived, electronically excited state, the corresponding application to photochromic photoreceptors would involve light-driven transitions between comparatively long-lived electronic ground states; hence, we expect that much less light power for both the activating and the deactivating light pulses will be needed than in STED. To our knowledge experiments along these lines have not been realized yet, but if they prove feasible, they may well enable “super-resolution optogenetics.”

Light-driven, bidirectional and reversible toggling between dark-adapted and signaling states makes photochromic photoreceptors also attractive for applications beyond their use as light-regulated actuators. Crucially, the two states not only differ in their biological activity but also in several other parameters including absorption and fluorescence properties. Photochromic photoreceptors might hence be developed into photoswitchable or photoactivatable fluorescent proteins.

## Fluorescent photoreceptors

Sensory photoreceptors are generally geared toward efficient photochemistry so as to achieve high sensitivity for light perception, cf. Section Photoreceptor Fundamentals; by contrast, the quantum yield for competing fluorescence processes is generally low. If, however, canonical photochemistry is impaired, e.g., via mutagenesis or protein truncation, fluorescence quantum yields can be greatly increased, and photoreceptors can thus be turned into efficient fluorescent proteins (FP), as demonstrated for LOV proteins (Drepper et al., 2007; Chapman et al., 2008) and bacterial phytochromes (Piatkevich et al., 2013a; Shcherbakova et al., 2015). Notably, the opposite route of converting FPs into sensory photoreceptors has been taken for the Dronpa protein (cf. Section Associating Photoreceptors and Optogenetic Applications) (Zhou et al., 2012). Since photoreceptor-derived FPs share with natural ones the key property of genetic encoding, they can be used as versatile reporters in much the same way as conventional FPs, provided their chromophores are available to sufficient extent. A broad palette of naturally occurring FPs notwithstanding, there are several use cases where FPs derived from sensory photoreceptors offer particular benefits.

First, LOV domains, which are rendered fluorescent by removal of a conserved cysteine residue required for canonical photochemistry (Drepper et al., 2007; Chapman et al., 2008), have a size of about 120 residues and are thus significantly smaller than the GFP-type jellyfish FPs (~220 residues). This size difference can be decisive in certain biological applications, e.g., those relying on transfection with viruses that have stringent limits on their genome size (Chapman et al., 2008; Konermann et al., 2013). Second, in contrast to jellyfish FPs, photoreceptor-derived FPs do not undergo slow maturation processes but become fluorescent once they incorporate their chromophore. Moreover, maturation of conventional FPs depends on molecular oxygen, making them less suited for studies under anoxic or low-oxygen conditions than photoreceptor-derived FPs which readily incorporate their chromophores in the absence of oxygen (Chapman et al., 2008). However, we caution that both the flavin-nucleotide and linear-tetrapyrrole chromophores used by LOV domains and bacteriophytochromes, respectively, are oxidized compounds and may hence not be present in sufficient amounts at very low oxygen concentrations. Further note that in the presence of oxygen, LOV domains lacking the conserved cysteine residue not only show increased fluorescence, but also they become efficient photosensitizers for generation of singlet-oxygen species. Although this property can deliberately be exploited, e.g., for blue-light-driven cell ablation (Qi et al., 2012), it may cause inadvertent effects in LOV fluorescence applications. Third, fluorescent reporters derived from bacteriophytochromes provide excitation and emission at longer wavelengths than can be achieved with conventional FPs (Shu et al., 2009; Filonov et al., 2011; Piatkevich et al., 2013a; Shcherbakova et al., 2015). Of particular relevance, bacteriophytochromes have been engineered that absorb and fluoresce within the near-infrared window of the electromagnetic spectrum (cf. Figure 1). In this wavelength regime, high penetration depths of light are afforded, and applications in deep tissue are enabled (cf. Section Photoreceptor Fundamentals). Fourth, photoreceptor-derived FPs not only serve as static markers but also as dynamic sensors for protein-protein interactions and certain cellular parameters (Shcherbakova et al., 2015). Specifically, the pumping activity in light-driven rhodopsin proton pumps has been disrupted by mutagenesis to yield weakly fluorescent proteins that report on membrane potential (Kralj et al., 2011, 2012). The resultant fully genetically encodable, voltage sensors have been used to visualize spontaneous membrane-voltage fluctuations in *E. coli* (Kralj et al., 2011) as well as action potentials in neuronal cells (Kralj et al., 2012). Fifth, sensory photoreceptors could prove particularly useful in the design of photoactivatable/photoswitchable FPs (Patterson and Lippincott-Schwartz, 2002). Notably, photoswitching is an inherent property of all photoreceptors, since light absorption drives transitions between dark-adapted and signaling states (plus additional states that may occur in the photocycle). In case of photochromic photoreceptors (cf. Section Photochromic Photoreceptors), state transitions can even be triggered in both directions by different colors of light. Since these states can considerably differ in their fluorescence properties (absorption and emission spectra, quantum yields), photochromic photoreceptors may be confectioned into versatile and efficient photoswitchable fluorescent probes. This has strikingly been demonstrated for certain bacteriophytochrome variants that serve as photoactivated, near-infrared fluorescent proteins (Piatkevich et al., 2013b).

For space constraints, we can but skim the engineering and fascinating applications of fluorescent photoreceptors; for in-depth coverage we recommend two recent review articles (Piatkevich et al., 2013a; Shcherbakova et al., 2015). The combination of sensory photoreceptors with fluorescent proteins, be it natural proteins, be it re-engineered proteins, augurs all-optical experiments in which both perturbation and readout are conducted optically. Since light-regulated actuators and fluorescent sensors are genetically encoded agents, such experiments can be conducted with the same level of reversibility, non-invasiveness and spatiotemporal definition as conventional optogenetic experiments. All-optical approaches should be particularly useful for closed-loop optogenetics where the behavior of a system is continuously monitored over time, and a measurable quantity, e.g., reporter fluorescence, is converted online into appropriate light pulses for optogenetic conditioning of said system (Paz et al., 2013). A particular impressive demonstration of the all-optical concept is provided by the combination of rhodopsin-based voltage sensors and light-gated ion channels that allow the simultaneous triggering and interrogation of action potentials in neurons (Hochbaum et al., 2014). Other biological processes, in particular cyclic-nucleotide metabolism, should also be amenable to all-optical approaches (Richter et al., 2015).

## Guidelines for photoreceptor engineering

The multitude of nifty applications in optogenetics impressively demonstrates the broad usefulness and general applicability of engineered photoreceptors. Although design presently proceeds on a case-by-case basis and often requires repeat trials before eventual success, powerful principles and recipes emerge that have proven particularly versatile and that may expedite future engineering efforts. In this chapter, we hence strive to distill insight gleaned from numerous specific examples into general guidelines for photoreceptor engineering. Of course, no single approach provides a panacea succeeding in each and every instance, but we hope the reader will find the below discussion illuminating. We subdivide this chapter into several interlinked, desirable traits in light-responsive systems and outline how they may be realized in photoreceptor engineering and optogenetic applications (Table 4).

**Table 4:** **Desiderata in photoreceptor engineering**.

**Aspect**	**Challenges and measures**
Design strategy	Most promising strategy to pursue? natural photoreceptor available associating photoreceptors (Section Associating Photoreceptors and Optogenetic Applications) order-disorder transitions (Section Light-regulated Order-disorder Transitions) homologous exchange of sensor modules (Section Light-regulated Tertiary and Quaternary Structural Transitions)
Dynamic range	Maximum activity difference dark vs. light? (Section Photoreceptor Fundamentals.) maximize free energy perturbation ΔΔG by choice of photosensor/effector, by linker optimization, by mutagenesis, by use of oligomeric photoreceptors minimize specific activity of T state and maximize specific activity of R state, e.g., by choice of effector, by mutagenesis embed photoreceptor in signaling networks that amplify response
Genetic encoding	Functional expression *in situ*? codon optimization cell-type-specific promoters intracellular trafficking signals ensure chromophore supply, e.g., by resorting to photoreceptors that use retinal, flavin-nucleotide and biliverdin chromophores
*In situ* activity	Appropriate activity levels *in situ*? adjust expression levels, especially for associating photoreceptors (Section Photoreceptor Fundamentals) vary specific activity by choice of effector module, by mutagenesis (e.g., attenuation of activity) embed photoreceptors in signaling networks for amplification of response
Light sensitivity	Can photoreceptor be activated to sufficient extent *in situ*? increase light power, improve light delivery use photoreceptors sensitive to long wavelengths at which light penetrates tissue more deeply embed photoreceptor in signaling networks for response amplification modulate effective light sensitivity at photostationary state by variation of dark-recovery kinetics (Section Photoreceptor Fundamentals)
Temporal resolution	Are the response kinetics sufficiently fast? accelerate *on*-kinetics by increasing light power, by signal amplification (so that activation of fewer photoreceptors accelerate *off*-kinetics by choice of photosensor, by speeding up dark-recovery reaction via mutagenesis, by speeding up downstream biological processes use photochromic photoreceptors for temporal depletion of signaling state (Section Photochromic Photoreceptors)
Spatial resolution	How can spatial resolution be improved? cell-type-specific expression and subcellular trafficking (Section Genetic Encoding) spatially restricted illumination use photochromic photoreceptors for spatial depletion of signaling state (Section Photochromic Photoreceptors)
Orthogonality	Parallel use of several photoreceptors and fluorescent proteins? selective excitation via spectral separation selective excitation via different light sensitivities selective excitation via different recovery kinetics use photochromic photoreceptors to counteract inadvertent cross-talk between excitation channels

### Design strategy

Arguably, the foremost consideration in photoreceptor engineering is that of eventual success: will arduous work finally bear fruit, and which is the most promising strategy to establish the desired light-responsive system? The sheer diversity of the above case studies strikingly illustrates that the choice of the most appropriate strategy is intricately linked to the identities of the desired sensor input and effector output. As a corollary, there is no single solution that guarantees success in each and every scenario; in this section, we hence draw general inferences from current examples, rather than treating individual cases for which we refer to Section Allostery of Photoreceptors and literature cited therein. In the following, we present recurring strategies that have been particularly successful in photoreceptor engineering and optogenetics (also cf. Table 4).

**Figure 6:**
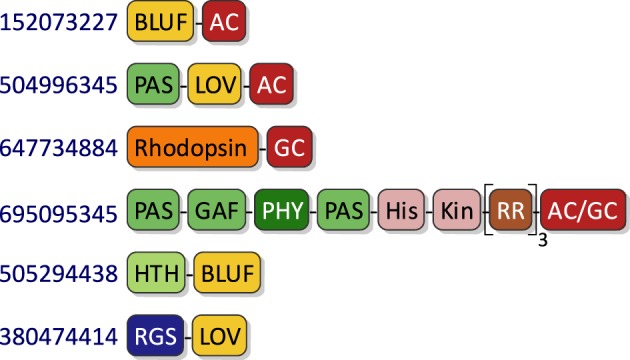
**Natural photoreceptors as optogenetic tools**. Certain naturally occurring photoreceptors can serve as powerful light-regulated actuators in optogenetics without any need for re-engineering. The diagram shows domain architectures of select sensory photoreceptors that have been deployed for optogenetics or that could be used to that effect; exemplary Genbank accession numbers are provided that possess certain architectures. Abbreviations as in main text and as follows: AC, adenylate cyclase; GC, guanlyate cyclase; HTH, helix-turn-helix; RGS, regulator of G-protein signaling; RR, response regulator.

By and large, the further one departs from natural systems (or previously engineered ones), the more challenging engineering and optogenetic applications become. By that token, in an ideal scenario, a natural photoreceptor exists that already exerts the demanded light-regulated biological activity and that can hence be used in optogenetics without any modification. Notable representatives are the light-gated channelrhodopsins (Nagel et al., 2002, 2003), certain animal rhodopsins (Oh et al., 2010; Spoida et al., 2014) and several light-activated nucleotide cyclases based on BLUF, LOV and rhodopsin photosensors (Schröder-Lang et al., 2007; Ryu et al., 2010; Stierl et al., 2011; Raffelberg et al., 2013; Avelar et al., 2014). Given the vast amounts of genome data (becoming) available, additional protein architectures of immediate optogenetic utility may be discovered in future (Figure 6).

In the absence of suitable natural photoreceptors, the most predictable and successful strategies in engineering light-responsive systems are based on light-dependent association reactions (cf. Section Associating Photoreceptors and Optogenetic Applications). Crucially, light-dependent recruitment and colocalization provide clear design rationales, especially when the target effector modules are naturally regulated via oligomerization processes. Strategies based on light-dependent association readily extend to split proteins where biological activity is only regained once the two halves are brought into proximity. Structural requirements of the linker between photosensor and effector modules are minimal, greatly facilitating photoreceptor engineering (cf. Section Associating Photoreceptors and Optogenetic Applications). Moreover, concrete design templates are provided by an ample body of literature on regulation of biological processes via chemically induced association. On the downside, light-regulated association reactions strongly depend on photoreceptor concentration which needs to be taken into account in applications (cf. Section Thermodynamics of Photoreceptors and Genetic Encoding). The relative performance of different associating photoreceptors may vary depending upon application context, and ideally several should be tested to achieve optimal optogenetic response.

Another class of highly versatile strategies for photoreceptor engineering capitalize on the light-triggered unfolding of the helical Jα appendage in the *As*LOV2 domain (Harper et al., 2003), where, again, a relatively clear design rationale is given (cf. Section Light-regulated Order-disorder Transitions). Interestingly, light-regulated Jα unfolding has been tapped in quite different, creative ways, e.g., to control the folding of covalently linked effector domains (Strickland et al., 2008), to control conformation and accessibility of peptide epitopes (Renicke et al., 2013; Bonger et al., 2014), or to control protein association (Lungu et al., 2012; Strickland et al., 2012). Often, the precise linker length and sequence have to be optimized to achieve efficient light regulation (e.g., Strickland et al., 2008; Wu et al., 2009). Moreover, several mutations have been identified that modulate the order-disorder equilibrium and that can thus be used to optimize the dynamic range of the light response (cf. Section Light-regulated Order-disorder Transitions). In general, light-regulated unfolding reactions are not specific to *As*LOV2 but represent a more general aspect of sensory photoreceptors as for example manifest in PYP. Consequently, similar engineering approaches may be implemented on the basis of photosensors other than *As*LOV2 and related phototropin LOV domains.

A last class of reasonably successful engineering strategies involve the recombination of homologous (photo)sensor and effector modules (e.g., Kim et al., 2005; Möglich et al., 2009a; Gasser et al., 2014) (cf. Section Light-regulated Tertiary and Quaternary Structural Transitions). As a design rationale, one exploits that sequence and structural homology between these modules often entails functional and mechanistic correspondence. However, this correspondence may not necessarily be a given, and several modules and design architectures may have to be tested to ensure eventual success. Moreover, in the first place, this engineering strategy depends on the availability of suitable homologous signal receptors and therefore appears less versatile than the above approaches based on light-dependent association and order-disorder transitions. Within this class, requirements on linker sequence and length are more demanding, as the linker often adopts defined structure and plays a critical role in governing overall activity, signal polarity and dynamic range (cf. Section Light-regulated Tertiary and Quaternary Structural Transitions).

At the end of this section, we reiterate that the selection of the best engineering strategy is tightly correlated with the identities of sensor and effector. Regardless of the precise strategy, all approaches benefit from efficient functional assays that allow the construction and screening of many candidate designs as well as the incremental improvement of functional designs. In particular, several studies demonstrate that the combination of random mutagenesis and high-throughput screening, often *in vivo*, can efficiently identify photoreceptor variants with altered and improved properties (e.g., Christie et al., 2007; Gleichmann et al., 2013; Ryu et al., 2014; Taslimi et al., 2014). These and related screening setups will doubtless be instrumental in photoreceptor engineering approaches that depart from the three categories listed above.

### Dynamic range

A key characteristic of sensory photoreceptors is their dynamic range for light regulation, i.e., the factor difference in activity between the dark-adapted and signaling states (cf. Section Thermodynamics of Photoreceptors). As illustrated in Figure 2, dynamic range is governed by both the amount of free energy that can be derived from incident light and the intrinsic activities *a*_R_ and *a*_T_ of the R and T states of the receptor. Notably, the maximum dynamic range that can be achieved is limited by the ratio of *a*_R_/*a*_T_ and by how well activity can be shut down in the low-activity state T, i.e., in the dark state for light-activated photoreceptors. Visible light has an energy content of a few hundred kJ per mol of absorbed photons which in principle would suffice to shift the equilibrium between T and R by many orders of magnitude. However, the amount of this energy that can be translated into relevant free energy changes is actually far less, for example about 16 kJ mol^−1^ for *As*LOV2 (Yao et al., 2008). Due to a pervasive lack of detailed biophysical data, neither is it known which value this parameter assumes in other photoreceptors nor how it could be modified (improved). Depending on the specific photoreceptor system, the intrinsic activities of T and R might be modulated via variation of photosensor and effector. For example, biological activity may be attenuated by homologous exchange of one effector module for another with different specific activity or by mutagenesis near the active site. Moreover, dynamic range can be improved by embedding (engineered) photoreceptors in downstream cellular signaling networks such that the light response is enhanced. Notably, amplification mechanisms of this kind are integral parts of cellular signaling, e.g., in gene-expression networks or in pathways involving second messengers (Stierl et al., 2011; Gasser et al., 2014; Jansen et al., 2015), and can be co-opted for optogenetic intervention. Furthermore, for oligomeric photoreceptors formation of the R state may be cooperative and may require the concomitant population of the signaling state in several photosensor domains, as illustrated in Figure 2F for associating photoreceptors and experimentally observed in the dimeric histidine kinase YF1 where both LOV subunits need to reside in their dark-adapted states to obtain full effector output (Möglich et al., 2009a). By this means, a larger free energy difference could be derived from light than achievable for a sole photosensor module. Finally, a more specific mechanism for amplification is realized in histidine kinases, like YF1, which catalyze opposing elementary phosphorylation and phosphatase reactions; since the biological response is governed by the net balance of these reactions, relatively small changes in the respective individual velocities may elicit disproportionally large net effects.

### Genetic encoding

To enable optogenetic perturbation of cells and tissues, photoreceptors have to be functionally expressed *in situ*. In many organisms, cell-specific promoters are known that restrict gene expression to a select set of cells or even to individual cells. In addition, it is desirable to direct photoreceptors to specific cellular compartments and organelles which could be accomplished via targeting sequences or fusion with (inert) proteins that normally reside at these sites (Zalocusky et al., 2013). As another necessary requirement, the chromophore cofactor of photoreceptors has to be available in sufficient quantities in the target tissue, and it has to be incorporated autonomously. Although the latter is generally true, the former is not necessarily given. Apparently, flavin nucleotides, retinal and the linear tetrapyrrole biliverdin widely occur across different cells and organisms (cf. Section Photoreceptor Fundamentals). By contrast, the tetrapyrrole phycocyanobilin utilized by plant phytochromes and cyanobacteriochromes is generally not available in most cells; it either needs to be added exogenously or produced endogenously by additionally introduced heterologous enzymes.

### *In situ* activity

Another important consideration is the overall activity levels of sensory photoreceptors *in situ* which are determined by expression levels and specific activity. Depending upon application, overall activity has to be adjusted to certain ranges to achieve relevant light-induced effects. If several photoreceptor systems with congruent functionality are available, as for light-activated adenylate cyclases (Schröder-Lang et al., 2007; Ryu et al., 2010; Stierl et al., 2011; Raffelberg et al., 2013; Avelar et al., 2014), one may select according to the specific activity of these systems. As outlined above, photoreceptors can be integrated into downstream signaling cascades so as to modulate or amplify the light effect. Expression and activity levels are of particular relevance for associating photoreceptors since their behavior and light response can vastly differ between concentration regimes (cf. Section Thermodynamics of Photoreceptors and Associating Photoreceptors and Optogenetic Applications and Figure 2F).

### Light sensitivity

Light delivery and light sensitivity are closely linked aspects in optogenetic applications. Depending upon the quality (color) and quantity (dose) required, light delivery to target sites *in situ* can be challenging, especially where opaque tissues and entire organs or animals are concerned. Short of increasing light power which is subject to demanding technical and biological limitations, light sensitivity may be optimized regarding both color and dose. Certain photoreceptors, namely phytochromes and cyanobacteriochromes, can be sensitive to red and infra-red light which penetrates tissue more deeply than light of shorter wavelengths. Especially within the “near-infra-red window,” lower light powers may hence suffice in optogenetic experiments (cf. Figure 1). To further decrease required light powers, the light response could be amplified in downstream signaling networks (cf. Section Dynamic Range), or the light sensitivity could be increased. Many optogenetic experiments are conducted at photostationary conditions, where individual photoreceptors repeatedly shuttle between their dark-adapted and signaling states, D and S, respectively. As discussed in Section Photoreceptor Fundamentals, in this regime, the effective light sensitivity is not only governed by the absolute sensitivity, i.e., the kinetics of going from D to S, but also by the reverse kinetics of going from S to D (cf. Figure 2A). The absolute light sensitivity can at best be modulated within rather narrow ranges; in particular, there is very limited scope for further enhancement since the quantum yields for photoreception are intrinsically high and cannot be increased beyond unity. By contrast, at least for some photoreceptor classes (phytochromes, LOV and rhodopsins) the reverse reaction and hence the effective light sensitivity can be varied by mutation across many orders of magnitude, cf., e.g., (Berndt et al., 2009), albeit at the cost of simultaneously affecting *off*-kinetics.

### Temporal resolution

Many optogenetic applications necessitate high temporal precision of both the *on*- and *off*-kinetics of the light response. As discussed in Section Photoreceptor Fundamentals, formation of the signaling state of photoreceptors after photon absorption occurs on the microseconds or faster timescale and hence is not rate-limiting for most biological scenarios. Rather, *on*-kinetics are limited by light delivery *in situ*, by the number of photoreceptors that need to assume their signaling state for triggering downstream signaling, and by ensuing biological processes that may be slow, e.g., gene expression. Accordingly, to accelerate *on*-kinetics, light power could be increased, the biological response could be amplified so that fewer photoreceptors need to assume their signaling state, and, in some cases, downstream biological processes could conceivably be accelerated. By contrast, *off*-kinetics are limited by the intrinsic dark-recovery reaction that returns the photoreceptor to its resting state, and by biological processes that may be inherently slow. Often, dark-recovery rates are strongly temperature-dependent and may hence become limiting if temperature is lowered. At least for LOV, rhodopsin and phytochrome photoreceptors, mutations are documented that can be introduced to deliberately modulate the recovery kinetics, although with two caveats: first, a change in dark-recovery kinetics will invariably affect the effective light sensitivity at photostationary state (cf. Section Photoreceptor Fundamentals and Light Sensitivity); second, these mutations should not be used indiscriminately as they could impair signal transduction (Diensthuber et al., 2014). Photochromic photoreceptors offer clear advantages in achieving fast *off*-kinetics since the return of the signaling state to the dark-adapted state of the photoreceptor can actively be driven by light (cf. Section Photochromic Photoreceptors).

### Spatial resolution

As another key advantage, optogenetics grants superior spatial resolution over competing perturbational approaches. One layer of spatial control is commonly achieved by cell-specific expression of sensory photoreceptors so that light sensitivity is confined to desired areas (cf. Section Genetic Encoding). Another layer is provided by spatially confined illumination so that photoreceptor activation is locally restricted, as impressively shown for, e.g., PA-Rac1 (Wu et al., 2009). Photochromic photoreceptors should be well-suited for achieving high spatial resolution in optogenetics since they can be bidirectionally toggled between states of different biological activity (e.g., Levskaya et al., 2009, and cf. Section Properties of Photochromic Photoreceptors). Crucially, not only can illumination thus be used to define areas of photoreceptor activation, but also areas of photoreceptor deactivation can be specified. For example, enhanced spatial resolution could benefit the optical targeting of subcellular compartments and of individual cells within eukaryotic tissues or microbial agglomerates.

### Orthogonality

If several sensory photoreceptors are deployed in parallel or in conjunction with fluorescent reporters (cf. Section Fluorescent Photoreceptors), it is imperative that light activation occur in an orthogonal manner, i.e., without inadvertent activation of other processes. Such orthogonality can be accomplished by spectral separation of light-responsive systems, e.g., by combining photoreceptors that are activated by blue light with fluorescent reporters that are monitored using red light. Generally, it appears favorable to resort to FPs sensitive to longer wavelengths than used for photoreceptor activation; notably, photoreceptors may be maximally sensitive to a relatively long wavelength, e.g., to red light in case of phytochromes, but to some extent they will be excited by all shorter wavelengths as well, e.g., by blue light. Again, photochromic photoreceptors offer unique advantages in that both formation and depletion of the signaling state can be controlled by light of different colors (cf. Section Photochromic Photoreceptors). If spectral separation is not feasible, orthogonality may still be achieved by resorting to systems that significantly differ in their light sensitivity, at the level of either the sensory photoreceptor or the associated downstream biological response. In a similar vein, separation may be possible in the kinetic domain; if two light-responsive systems strongly differ in dark-recovery kinetics, selective activation of the system with the slower recovery could be effected by pulsed illumination. These concepts are exemplified by the recently discovered channelrhodopsin variants Chronos and Chrimson (Klapoetke et al., 2014; Schmidt and Cho, 2015): Chrimson can be selectively excited with red light which Chronos does not respond to; *vice versa*, Chronos can be selectively excited with defined intensities of blue light as it is much more sensitive (in terms of photocurrents) than Chrimson which also responds to blue light, albeit to much lower extent. Moreover, Chronos has much faster response kinetics than Chrimson which allows kinetic separation (Schmidt and Cho, 2015). Given the continuing success of optogenetics and the emergence of all-optical approaches (Hochbaum et al., 2014; Richter et al., 2015), we expect orthogonal activation of sensory photoreceptors to become increasingly relevant.

## Conclusion

Owing to tremendous advances over the recent past, optogenetics now constitutes a general, highly versatile and readily implementable approach for the perturbation and quantitative analysis of diverse cellular processes across the neurosciences and biology. Whereas initially optogenetics and photoreceptor engineering had been the province of specialists, by now they have well moved into the domain of applicants interrogating specific cellular and neural circuits. This welcome development is doubtless aided by the availability of a rich and continuously expanding toolkit of natural and engineered light-regulated actuators. At the same time, the emergence of particularly successful and reusable templates for photoreceptor engineering reviewed here (cf. Section Allostery of Photoreceptors and Guidelines for Photoreceptor Engineering) further promotes the wider adoption of optogenetics. Currently deployed optogenetic actuators mostly target general, broad-range processes as gene expression, membrane potential changes, protein-protein interactions, protein degradation or second-messenger metabolism. If, however, photoreceptor engineering further improves and simplifies to the extent where it becomes essentially predictable, more specific, narrow-range processes, e.g., the activity of particular protein kinases with just a few substrates, become worthwhile targets for optogenetic intervention. Beyond optogenetics, we note the recent emergence of conceptually similar techniques for the spatiotemporally defined, non-invasive and reversible perturbation of cellular events that rely on application of radio waves and alternating magnetic fields instead of light (Stanley et al., 2012, 2015; Leibiger and Berggren, 2015). These so-called radiogenetic and magnetogenetic strategies (Leibiger and Berggren, 2015) could well supplement existing and future optogenetic approaches.

### Conflict of interest statement

The authors declare that the research was conducted in the absence of any commercial or financial relationships that could be construed as a potential conflict of interest.

## Abbreviations

BLUF: sensors of blue light using flavin adenine dinucleotide
BV: biliverdin
cAMP: 3′,5′-cyclic adenosine monophosphate
CBCR: cyanobacteriochrome
cGMP: 3′,5′-cyclic guanosine monophosphate
FAD: flavin adenine dinucleotide
FMN: flavin mononucleotide
FP: fluorescent protein
GAF: cGMP-specific phosphodiesterases, adenylate cyclases and FhlA
GPCR: G-protein-coupled receptor
LOV: light-oxygen-voltage
MAP kinase: mitogen-activated protein kinase
MTHF: methenyltetrahydrofolate
PAS: Per-ARNT-Sim
pCA: p-coumaric acid
PCB: phycocyanobilin
PΦB: phytochromobilin
Phy: phytochrome
PHY: phytochrome-specific domain
PYP: photoactive yellow protein (xanthopsin)
RTK: receptor tyrosine kinase
STED: stimulated emission depletion.
